# Towards Predicting Partitioning of Enzymes between Macromolecular Phases: Effects of Polydispersity on the Phase Behavior of Nonadditive Hard Spheres in Solution †

**DOI:** 10.3390/molecules27196354

**Published:** 2022-09-26

**Authors:** Luka Sturtewagen, Erik van der Linden

**Affiliations:** Laboratory of Physics and Physical Chemistry of Foods, Wageningen University, Bornse Weilanden 9, 6708 WG Wageningen, The Netherlands

**Keywords:** polydispersity, hard spheres, phase behavior, virial coefficient

## Abstract

The ability to separate enzymes, or cells or viruses, from a mixture is important and can be realized by the incorporation of the mixture into a macromolecular solution. This incorporation may lead to a spontaneous phase separation, with one phase containing the majority of one of the species of interest. Inspired by this phenomenon, we studied the theoretical phase behavior of a model system composed of an asymmetric binary mixture of hard spheres, of which the smaller component was monodisperse and the larger component was polydisperse. The interactions were modeled in terms of the second virial coefficient and could be additive hard sphere (HS) or nonadditive hard sphere (NAHS) interactions. The polydisperse component was subdivided into two subcomponents and had an average size ten or three times the size of the monodisperse component. We gave the set of equations that defined the phase diagram for mixtures with more than two components in a solvent. We calculated the theoretical liquid–liquid phase separation boundary for the two-phase separation (the binodal) and three-phase separation, the plait point, and the spinodal. We varied the distribution of the polydisperse component in skewness and polydispersity, and we varied the nonadditivity between the subcomponents as well as between the main components. We compared the phase behavior of the polydisperse mixtures with binary monodisperse mixtures for the same average size and binary monodisperse mixtures for the same effective interaction. We found that when the compatibility between the polydisperse subcomponents decreased, the three-phase separation became possible. The shape and position of the phase boundary was dependent on the nonadditivity between the subcomponents as well as their size distribution. We conclude that it is the phase enriched in the polydisperse component that demixes into an additional phase when the incompatibility between the subcomponents increases.

## 1. Introduction

The ability to separate enzymes or other compounds such as cells or viruses from their mixture is important. This separation can be realized by the incorporation of the mixture into a polymeric solution after which a spontaneous asymmetric partitioning over two macromolecular solutions may occur, where the majority of the compound of interest resides in one of the phases [1]. For modeling such a separation, we consider mixing two different types of macromolecules in an aqueous phase. The demixing depends on the polydispersity of the macromolecules. Regarding separating enzymes and similar compounds, the prediction of the phase separation is important. Apart from such applications, the separation of aqueous phases within the cytoplasm has also received interest [2]. Interestingly, the preassembly mechanism during evolution [3] may be speculatively related to the same separation mechanisms. In the study of the phase behavior of binary mixtures, the components are usually assumed to be pure and monodisperse; however, in nature, most components are not that neatly monodisperse. Many components show a size and charge variation or contain hard-to-remove particles that can influence their phase behavior in binary mixtures. In experimental work, Sager [4] reported that even small impurities can lead to drastic shifts in the position of the phase boundary. Moreover, the compatibility between components can depend on the temperature [5], salt concentration or pH of the solution [6].

To learn more about the underlying mechanisms regarding the separation of enzymes, for example, from another macromolecular compound, where both are in an aqueous phase, one may, to a first approximation, model these molecules as spheres. In a simplified picture, the interactions can be distinguished to emerge from two different physical mechanisms. The first one involves only excluded volume interactions between the spheres. In this mechanism, the minimal distance between the particles is determined by the sum of their respective radii [7]. This is the so called additive hard sphere interaction (HS). With this mechanism, the phase separation is driven by a size asymmetry between the particle sizes [8]. This asymmetry leads to the depletion of small spheres around the large spheres and as a result, to an effective attraction (depletion interaction) between the larger spheres [9]. For more information on depletion interactions, we refer to a book by Lekkerkerker and Tuinier [10]. We note that the solvent molecules present are still much smaller than the spheres under consideration and are effectively integrated out of the analysis. The other mechanism is when the distance between the particles of a different species can be larger or smaller than the sum of their respective radii. This is referred to as nonadditive hard sphere (NAHS) interaction. Previous research has shown that already at small degrees of nonadditivity, it becomes possible for components with no size asymmetry to demix [11,12]. Either way, upon phase separation, the mixture will demix into two (or more) phases, each enriched in one of the components. In a previous article, we focused on the first type of interaction [13]. We investigated the influence of size polydispersity on the phase behavior of an additive binary asymmetric mixture. In this work, we focus on the second type, binary (polydisperse) mixtures where the distance between the particles of different species can be larger or smaller than the sum of their respective radii.

Piech and Walz [14] studied the effect of size polydispersity and charge heterogeneity on the depletion interaction in a colloidal system. They found that the size distribution in the larger particle had a different effect on the depletion attraction for charged and noncharged hard sphere systems, for the depletion attraction decreased between the larger particles at constant volume fraction due to the polydispersity. This effect was further enhanced by the presence of a charge. Polydispersity significantly lowered the magnitude of the repulsive barrier.

The nonadditivity is usually described by the nonadditivity parameter Δ (with Δ≥−1). When Δ=0, the mixture has an additive hard sphere interaction, and the closest approach of the particles is the sum of their radii. When Δ<0, the two particles experience more attraction and can come closer to each other than the sum of their radii, while when Δ>0, the two particles have more repulsion, and their distance of closest approach is larger than the sum of their respective radii. It is clear that this can have enormous effects on their phase behavior. Particles with a negative Δ tend to be more compatible with each other, while particles with a positive Δ are less compatible and tend to demix at lower concentrations. Already at the relatively low Δ=0.1, it becomes possible for components with the same size to demix [15].

Paricaud [16] studied the phase behavior of polydisperse colloidal dispersions. Their mixture consisted of a monodisperse component and a polydisperse component. The interaction between the monodisperse and polydisperse components was assumed to be NAHS (with the same Δ for all polydisperse spheres), while the interaction between the polydisperse components amongst themselves was assumed to be additive HS. They found that the critical point of a polydisperse mixture was at a lower solution pressure than for completely monodisperse mixtures. For mixtures with a large variation in the size of the polydisperse mixtures, they observed the possibility of a three-phase system. The phase behavior of a colloid and a polydisperse polymer was studied by Sier and Frenkel [17]. They used the Asakura–Osawa model for the interactions between the different components. They found that increasing the polydispersity increased also the extent of the fluid–fluid coexistence. They reasoned that the introduction of larger polymer coils was the driving force towards phase separation.

In this study, we aim to get a better understanding of how nonadditive interaction influences the phase behavior of binary mixtures with some polydispersity or impurities. We study the position of the phase separation boundary, the spinodal, and the critical point. Moreover, we aim to predict the fractionation of the polydisperse component between the different phases. We model the interactions between the different components using the second virial coefficient (Section 2.1). In Section 2.2, we describe the equations for the spinodal; in Section 2.3, we describe the equations for the critical point; and finally, in Section 2.4, we describe the equations defining the phase boundary. With the expressions in Section 2, we have enough to calculate the phase diagram for a variety of mixtures described in Section 3. First, we introduce nonadditivity between the main components in the binary mixtures (Section 3.1); subsequently, we introduce nonadditivity between the subcomponents in the polydisperse component (Section 3.2); and finally, we combine both in Section 3.3. In Section 3.4, we look into the fractionation of some of the mixtures from Section 3.2 at a specific parent concentration.

## 2. Theory

We show the equations used for the calculations of the phase diagram of the different studied systems: the set of equations defining the stability boundary, the critical point, and phase boundaries of a mixture. All sets of equations were solved in Matlab R2017b. For a more detailed derivation of the equations, we refer to an earlier reference [13].

### 2.1. Osmotic Virial Coefficient

The osmotic pressure, Π, of a solution at a temperature *T*, can be written as a virial expansion, similar to the virial expansion of the universal gas law for real gasses [18]:

(1) $$\beta \Pi =\rho +B_{2}\left(T,\mu_{s}\right)\rho^{2}+B_{3}\left(T,\mu_{s}\right)\rho^{3}+...$$

with $\beta =\frac{1}{kT}$, *k* the Boltzmann’s constant, ρ the number density of the component $\left(\frac{N_{\nu }}{V}\right)$, $B_{2}$ the second virial coefficient, and $B_{3}$ the third virial coefficient. The second virial coefficient accounts for the increase in osmotic pressure due to particles’ pairwise interaction. The third virial coefficient accounts for the interaction between three particles in a variety of configurations. The equation can be expanded for higher densities with $B_{n}$, the nth virial coefficient, which accounts for the interaction between *n* different particles.

In this work, we limit the virial expansion to the second virial coefficient, which is given by [10]:

(2) $$B\left(T,\mu_{s}\right)=2\pi \int_{0}^{\infty }r^{2}\left(1-\exp\left[-\beta W(r)\right]\right)dr$$

in which $\mu_{s}$ is the chemical potential of the solution, W(r) is the interaction potential between the particles, and *r* is the distance.

For an additive hard sphere (HS) interaction, the interaction potential for two particles (of the same species or different species) is given by:

(3) $$W{\left(r\right)}_{HS}=\left\{\begin{array}{cc} 0, & r>\sigma_{ij}\\ \infty , & r\leq \sigma_{ij} \end{array}\right.$$

with $\sigma_{ij}=\left(\sigma_{i}+\sigma_{j}\right)/2$ the distance between the centers of the two particles.

For nonadditive hard spheres (NAHS), the distance of the closest approach of the centers of the two particles of different species can be closer or further than the distance between their centers [11]. The closest distance then becomes: $\sigma_{ij}=\left(\left(\sigma_{i}+\sigma_{j}\right)/2\right)\left(1+\Delta \right)$, in which Δ(≥−1) accounts for the nonadditivity of the interaction between the particles. When Δ>0, the distance of closest approach of both spheres increases and when Δ<0, the distance of closest approach decreases compared to that due to the HS interaction only. For the additive hard sphere interaction, Δ=0.

In a mixture with *n* distinguishable components in a solution, there are two main types of two-particle interactions that can occur: between particles of the same species and particles of different species.

For the second virial coefficient given by Equation (2), using the interaction potential defined in Equation (3), we find:

(4) $$\begin{array}{cc} B_{xx} & =\frac{2\pi }{3}{\left(\sigma_{x}\right)}^{3} \end{array}$$

(5) $$\begin{array}{cc} B_{xy} & =\frac{2\pi }{3}{\left(\left(\frac{\sigma_{x}+\sigma_{y}}{2}\right)\left(1+\Delta \right)\right)}^{3} \end{array}$$

where $B_{xx}$ is the second virial coefficient for particles of the same species (assumed to be HS) and $B_{xy}$ is the second virial coefficient of particles of different species, which can be HS or NAHS.

The general equation for the osmotic pressure for a dilute mixture is given by [13]:

(6) $$\begin{array}{cc} \beta \Pi & =\rho +B_{11}\rho_{1}^{2}+2B_{12}\rho_{1}\rho_{2}+2B_{13}\rho_{1}\rho_{3}...\\ \; & =\rho +\sum_{i}^{n}\sum_{j}^{n}B_{ij}\rho_{i}\rho_{j}+... \end{array}$$

**Figure 1:**
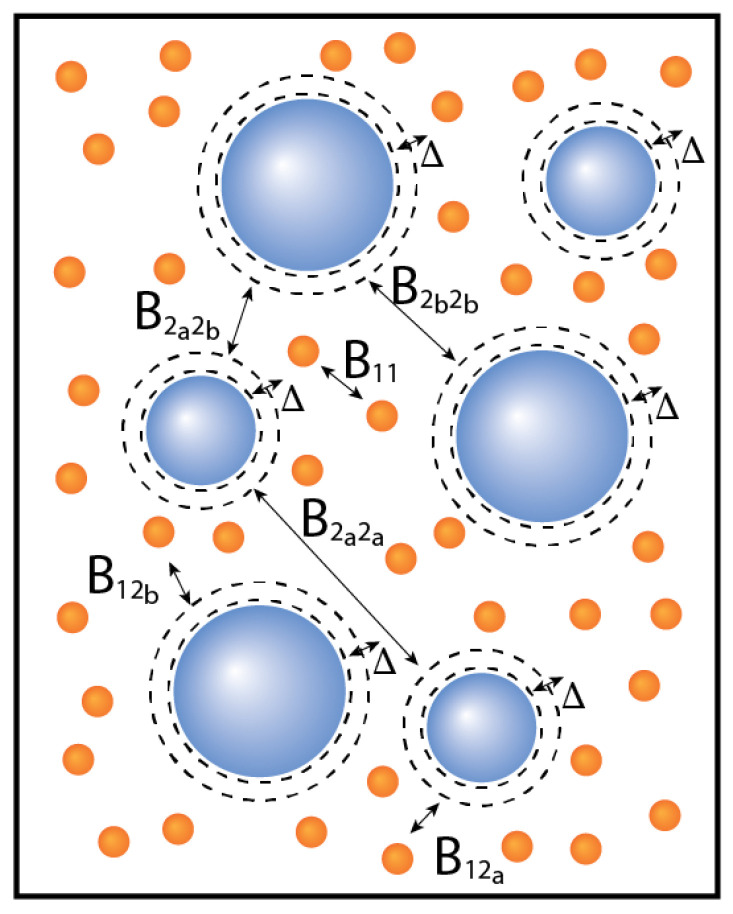
Graphical representation of a simple polydisperse mixture, in which the polydisperse component consists of two subcomponents (*a* and *b*, n=3); second virial coefficients are indicated. The distance of closest approach is influenced by Δ: Δ>0 increases this distance, Δ<0 decreases this distance.

In this article, we focus on binary mixtures in which one of the components consists of subcomponents (Figure 1). By increasing the number of subcomponents, the number of equations to solve for the phase diagram increases. Just as in the previous article [13], we also compare the results to the number average virial coefficients of the different components. The number average virial coefficient was chosen because it allows for a comparison to experiments, e.g., the virial coefficient obtained from osmometric measurements [19].

The number average second virial coefficient of a mixture can be written as:

(7) $$\begin{array}{cc} B_{mix} & =B_{11}x_{1}^{2}+2B_{12}x_{1}x_{2}+2B_{13}x_{1}x_{3}...\\ \; & =\sum_{i}^{m}\sum_{j}^{m}B_{ij}x_{i}x_{j} \end{array}$$

in which $B_{ii}$ is the second virial coefficient of the *i*th particle, $B_{ij}$ is the second cross virial coefficient of the *i*th particle and the *j*th particle, and $x_{i}$ is the fraction of the *i*th particle, $\sum x_{i}=1$.

Using this definition, we can map the binary mixture consisting of, for example, a monodisperse component 1 and a component 2 subdivided into two subcomponents (*a* and *b*) by a 2×2 matrix of virial coefficients. We refer to this 2×2 matrix as the effective virial coefficient matrix.

(8) $$\begin{array}{ccc} B_{11_{eff}} & = & B_{11}\\ B_{12_{eff}} & = & x_{a}B_{12_{a}}+x_{b}B_{12_{b}}\\ B_{22_{eff}} & = & x_{a}^{2}B_{2_{a}2_{a}}+2x_{a}x_{b}B_{2_{a}2_{b}}+x_{b}^{2}B_{2_{b}2_{b}} \end{array}$$

The effective virial coefficient matrix for this mixture then becomes:

(9) $$B_{eff}=\left[\begin{array}{cc} B_{11_{eff}} & B_{12_{eff}}\\ B_{12_{eff}} & B_{22_{eff}} \end{array}\right]$$

### 2.2. Stability of a Mixture

The stability of a mixture is dependent on the second derivative of the free energy. If the second derivative of the mixture becomes zero, the mixture is at the boundary of becoming unstable. Unstable mixtures have a negative second derivative [20,21].

The differential of the free energy of a mixture is given by [18]:

(10) $$dA=-SdT-pdV+\sum_{i}^{n}\mu_{i}dN_{i}$$

in which $\mu_{i}$ and the chemical potential (the first partial derivative of the free energy with respect to number of particles ($N_{i}$) for component *i* is given by:

(11) $$\mu_{i}=\mu_{i}^{0}+kT\ln\left(\rho_{i}\right)+2kT\left(\sum_{j}^{n}B_{ij}\rho_{j}\right)$$

For a mixture with *n* distinguishable components, the second partial derivatives can be represented by a n×n matrix of the first partial derivatives of the chemical potential of each component.

This results in the following general stability matrix:

(12) $$\begin{array}{cc} M_{1} & =\left[\begin{array}{ccc} \frac{\partial \mu_{1}}{\partial N_{1}} & \cdots & \frac{\partial \mu_{1}}{\partial N_{n}}\\ \vdots & \ddots & \vdots \\ \frac{\partial \mu_{n}}{\partial N_{1}} & \cdots & \frac{\partial \mu_{n}}{\partial N_{n}} \end{array}\right]\\ \; & =\left[\begin{array}{ccc} 2B_{11}+\frac{1}{\rho_{1}} & \cdots & 2B_{1n}\\ \vdots & \ddots & \vdots \\ 2B_{1n} & \cdots & 2B_{nn}+\frac{1}{\rho_{n}} \end{array}\right] \end{array}$$

The mixture is stable when all eigenvalues are positive [22]; when, on the other hand, one of the eigenvalues is not positive, the mixture becomes unstable. The limit of stability is reached when the matrix has one zero eigenvalue and is otherwise positive definite, and is referred to as the spinodal [23].

When there are only two components in the mixture (n=2), the spinodal is defined by the condition $\det M_{1}=0$. When the number of components is larger (n>2), $\det M_{1}=0$ can have more than one solution [22]. The spinodal can be found by checking whether $\det M_{1}$ changes sign for small changes in the concentrations of the components.

### 2.3. Critical Points

In a binary mixture, the critical point is a stable point which lies on the stability limit (spinodal) [23] and where the phase boundary and spinodal coincide. In mixtures of more components, these critical points become plait points. Critical points and plait points are in general concentrations at which two phases are in equilibrium and become indistinguishable [24].

There are two criteria that can be used to find critical points. The first one is $det(M_{1})=0$, which is the equation for the spinodal. The other criterion is based on the fact that at the critical point, the third derivative of the free energy should also be zero. For a multicomponent system, this criterion can be reformulated using Legendre transforms as $det(M_{2})=0$ [21,25], where:

(13) $$M_{2}=\left[\begin{array}{ccc} \frac{\partial \mu_{1}}{\partial N_{1}} & \cdots & \frac{\partial \mu_{n}}{\partial N_{n}}\\ \vdots & \ddots & \vdots \\ \frac{\partial M_{1}}{\partial N_{1}} & \cdots & \frac{\partial M_{1}}{\partial N_{n}} \end{array}\right]$$

Matrix $M_{2}$ is matrix $M_{1}$ with one of the rows replaced by the partial derivatives of the determinant of matrix $M_{1}$. Note: it does not matter which row of the matrix is replaced.

### 2.4. Phase Boundary

When a mixture becomes unstable and demixes into two or more phases, the chemical potential of each component and the osmotic pressure is the same in all phases [18].

(14) $$\left\{\begin{array}{ccc} \beta \Pi^{I} & =\beta \Pi^{II} & =\cdots \\ \beta \mu_{1}^{I} & =\beta \mu_{1}^{II} & =\cdots \\ \; & \vdots & \\ \beta \mu_{n}^{I} & =\beta \mu_{n}^{II} & =\cdots \end{array}\right.$$

where the phases are denoted by I,II,....

For a mixture containing *n* distinguishable components, which demixes into two phases, this results in n+1 equations and 2×n unknowns. If the mixture demixes into three phases, this results in 2×n+2 equations and 3×n unknowns. To solve the set of equations without having to fix the concentration of one component and the ratio between the other components for at least one of the phases, we need extra equations. For the extra set of equations, we build on the fact that no particles are lost and no new particles are created during phase separation, and the fact that we assume that the total volume does not change.

For a system that separates into three phases, we then obtain:

$$\rho =\sum_{i}^{n}\rho_{i}=\frac{\sum_{i}^{n}N_{i}}{V}=\frac{\sum_{i}^{n}N_{i}^{I}+\sum_{i}^{n}N_{i}^{II}+\sum_{i}^{n}N_{i}^{III}}{V^{I}+V^{II}+V^{III}}$$

which can be rewritten as [13]:

$$\begin{array}{cc} \rho & =\alpha^{I}\sum_{i}^{n}\rho_{i}^{I}+\alpha^{II}\sum_{i}^{n}\rho_{i}^{II}+\left(1-\alpha^{I}-\alpha^{II}\right)\sum_{i}^{n}\rho_{i}^{III} \end{array}$$

with

$$\alpha^{I}=\frac{V^{I}}{\sum_{i}^{f}V^{i}}$$

in which *f* denotes the number of phases.

This results in the following set of equations:

(15) $$\left\{\begin{array}{cc} \beta \Pi^{I} & =\beta \Pi^{II}=\cdots \\ \beta \mu_{1}^{I} & =\beta \mu_{1}^{II}=\cdots \\ \; & \vdots \\ \beta \mu_{n}^{I} & =\beta \mu_{n}^{II}=\cdots \\ \rho_{1} & =\alpha^{I}\rho_{1}^{I}+\cdots +\left(1-\sum_{i}^{f-1}\alpha^{i}\right)\rho_{1}^{f}\\ \; & \vdots \\ \rho_{n} & =\alpha^{I}\rho_{n}^{I}+\cdots +\left(1-\sum_{i}^{f-1}\alpha^{i}\right)\rho_{n}^{f} \end{array}\right.$$

With this set of equations, we have 2×n+1 unknowns and 2×n+1 equations for mixtures that separate into two phases. For mixtures that demix into three phases, we have 3×n+2 unknowns and 3×n+2 equations. Therefore, this set of equations allows the calculation of the concentration of each component in each of the phases for any given parent concentration, given that the mixture will demix at this concentration.

## 3. Results and Discussion

In this work, we calculated the phase diagram for a variety of binary nonadditive mixtures of a small hard sphere *A* and a larger hard sphere *B* with a size ratio $q=\sigma_{A}/\sigma_{B}$. Component *B* was subdivided into two subcomponents and was characterized by a degree in polydispersity (PD), defined by:

$$PD=\frac{\sqrt{\sum {\left(\sigma_{B_{i}}-\sigma_{B}\right)}^{2}\times N_{B_{i}}/N_{B}}}{\sigma_{B}}\times 100$$

We varied the nonadditivity between the particles of component *A* and *B* ($\Delta_{AB}$), and between the subcomponents of *B* ($\Delta_{B_{a}B_{b}}$). In addition, we varied the degree of polydispersity (PD) of component *B* and the distribution between the subcomponents as well as the size ratio (*q*) between components *A* and *B*.

For all particles, the concentrations were expressed as a dimensionless parameter according to $\eta =\frac{\pi \rho \sigma^{3}}{6}$. We calculated the critical point, the phase separation boundary, and the spinodal of the various mixtures. Moreover, we also investigated the composition of the child phases, volume fraction of the phases (α), and the fractionation of the polydisperse component *B* for a specific parent mixture (η=(0.010,0.200)), for mixtures with a size ratio $q=\sigma_{A}/\sigma_{B}=1/10$ and $\Delta_{AB}=0$, while varying the nonadditive interaction between the subcomponents of *B* ($\Delta_{B_{a}B_{b}}$).

### 3.1. Nonadditive Interaction between Components *A* and *B* ($\Delta_{AB}$)

**Figure 2:**
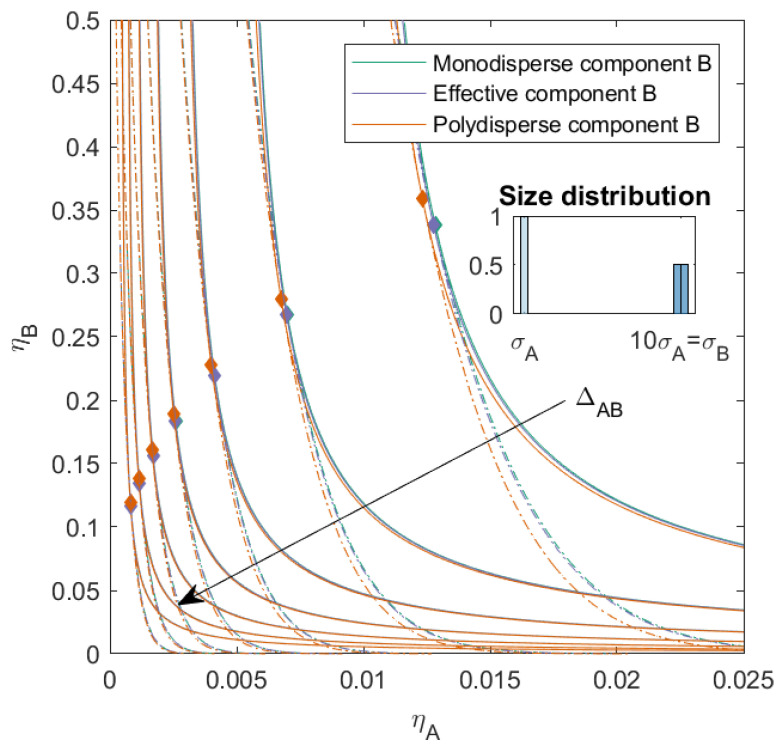
Phase diagram for binary (components *A* and *B*) nonadditive hard sphere mixture with size ratio $q=\sigma_{A}/\sigma_{B}=1/10$ plotted as a function of the partial packing fractions, $\eta_{A}$ and $\eta_{B}$; as indicated in the inset, component *A* is monodisperse, component *B* is polydisperse (PD=4.00). The symbols $\sigma_{A}$ and $\sigma_{B}$ refer to the diameters of species A and B, respectively. The interaction between components *A* and *B* is nonadditive, the nonadditivity parameter $\Delta_{AB}$ was varied from −0.1 to 0.5 with a step size of 0.1 (the arrow indicates increasing $\Delta_{AB}$). The interaction between the subcomponents of *B* is additive. The spinodal (solid line) and binodal (dashed line) meet each other at the critical point (diamond).

For the first set of mixtures (see Figure 2), we calculated the phase diagram for binary mixtures with a nonadditive interaction between monodisperse component *A* and slightly polydisperse component *B*, with two subcomponents and a PD=4.00. These two subcomponents were additive hard spheres in two sizes (both present in the same amount), with the number average size of the mixture equal to 10 times the size of component *A*. The mixture therefore consisted of three components of different sizes. We varied the nonadditivity between components *A* and *B* ($\Delta_{AB}$, the same for both subcomponents) from −0.1 to 0.5 with a step size of 0.1. When $\Delta_{AB}=0$, the interaction between all components was equivalent to an additive hard sphere interaction. We calculated the phase diagram using both the simplified 2×2 effective virial coefficient matrix described in the theory (we refer to this as the effective mixture *B*) and the full 3×3 virial coefficient matrix (to which we refer as the polydisperse mixture *B*). These mixtures were also compared to mixtures in which component *B* was monodisperse with a size equal to the average particle size of component *B* (we refer to this as the monodisperse mixture *B*).

With increasing $\Delta_{AB}$, the phase boundary, spinodal, and critical point shifted towards lower concentrations, for the monodisperse mixture, effective mixture, and polydisperse mixture. This was in line with research on nonadditive binary mixtures [26]. The difference between the phase boundary, spinodal, and critical point of the monodisperse mixture and the effective mixture was negligible, for all $\Delta_{AB}$. We saw however that the introduction of the polydispersity caused the critical point to shift to a higher volume fraction of component *B* and that, especially at a lower volume fraction of component *B*, the phase separation boundary shifted towards slightly lower packing fractions. This effect was more pronounced when $\Delta_{AB}$ was small.

When the PD of component *B* increased, or the distribution of the subcomponents of *B* varied, we saw the same pattern as in Figure 2 (see the Supplementary Materials). However, we see that, as discussed in [13], the critical point shifted towards higher concentrations of *B* for the polydisperse mixtures depending on the size and concentration of the largest subcomponent of *B*, and the difference between the effective and the monodisperse mixtures increased with the size of the largest subcomponent of *B*.

### 3.2. Nonadditive Interaction within Polydisperse Component *B* ($\Delta_{B_{a}B_{b}}$)

In the next set of mixtures, we kept the interaction between the components *A* and *B* as hard-sphere additive, but we introduced some nonadditivity in the interaction between the subcomponents of *B*. We varied $\Delta_{B_{a}B_{b}}$ from −0.10 to 0.10 with a step size of 0.05. When $\Delta_{B_{a}B_{b}}$ was small, the subcomponents were more compatible with each other; when, on the other hand, $\Delta_{B_{a}B_{b}}$ increased and became positive, the compatibility between the subcomponents decreased. When $\Delta_{B_{a}B_{b}}>0$, it became possible for components of similar size to phase-separate [26].

**Figure 3:**
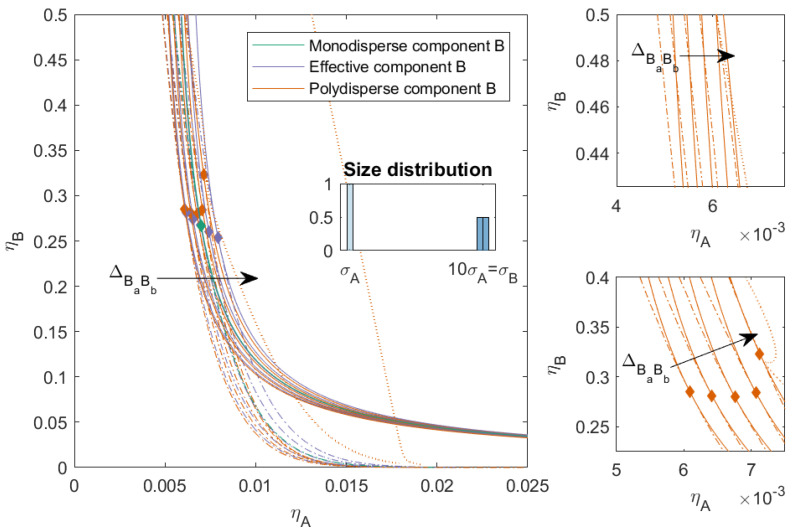
Phase diagram for binary (components *A* and *B*) nonadditive hard sphere mixture with size ratio $q=\sigma_{A}/\sigma_{B}=1/10$, plotted as a function of the partial packing fractions, $\eta_{A}$ and $\eta_{B}$; component *A* is monodisperse, component *B* is polydisperse (PD=4.00). The symbols $\sigma_{A}$ and $\sigma_{B}$ refer to the diameters of the species A and B, respectively. The interaction between components *A* and *B* is additive, the interaction between the subcomponents *B* is nonadditive, and the nonadditivity parameter $\Delta_{B_{a}B_{b}}$ was varied from −0.1 to 0.1 with a step size of 0.05 (the arrow indicates increasing $\Delta_{B_{a}B_{b}}$). The spinodal (solid line) and binodal (dashed line) meet each other at the plait point (diamond), the three-phase boundary is indicated with a dotted line and meets the spinodal at the plait point (diamond). The two right-hand smaller figures represent zoomed-in sections of the main figure.

In Figure 3, we plotted the phase diagram for the binary mixtures with PD=4.00 and $\Delta_{AB}=0$, and we varied $\Delta_{B_{a}B_{b}}$. When $\Delta_{B_{a}B_{b}}>0$, the compatibility between the subcomponents decreased and the phase separation into three phases became possible (depicted as the dotted line in the figure). Mixtures with a smaller $\Delta_{B_{a}B_{b}}$ demixed into two phases at lower concentrations compared to the completely hard sphere mixture. Mixtures with a larger $\Delta_{B_{a}B_{b}}$ demixed into two phases at higher packing fractions compared to the completely hard sphere mixture, and also had a three-phase boundary. The three-phase boundary was at lower concentrations for larger $\Delta_{B_{a}B_{b}}$ and came close to the two-phase boundary for the mixture with $\Delta_{B_{a}B_{b}}=0.10$. The critical point of the polydisperse mixtures changed depending on the nonadditivity of the subcomponents: the critical point was at its lowest concentrations of *A* for negative $\Delta_{B_{a}B_{b}}$, its lowest concentration of *B* when the interaction between the subcomponents of *B* became more like HS, and the concentration of the critical point for *B* increased with $\Delta_{B_{a}B_{b}}$.

**Figure 4:**
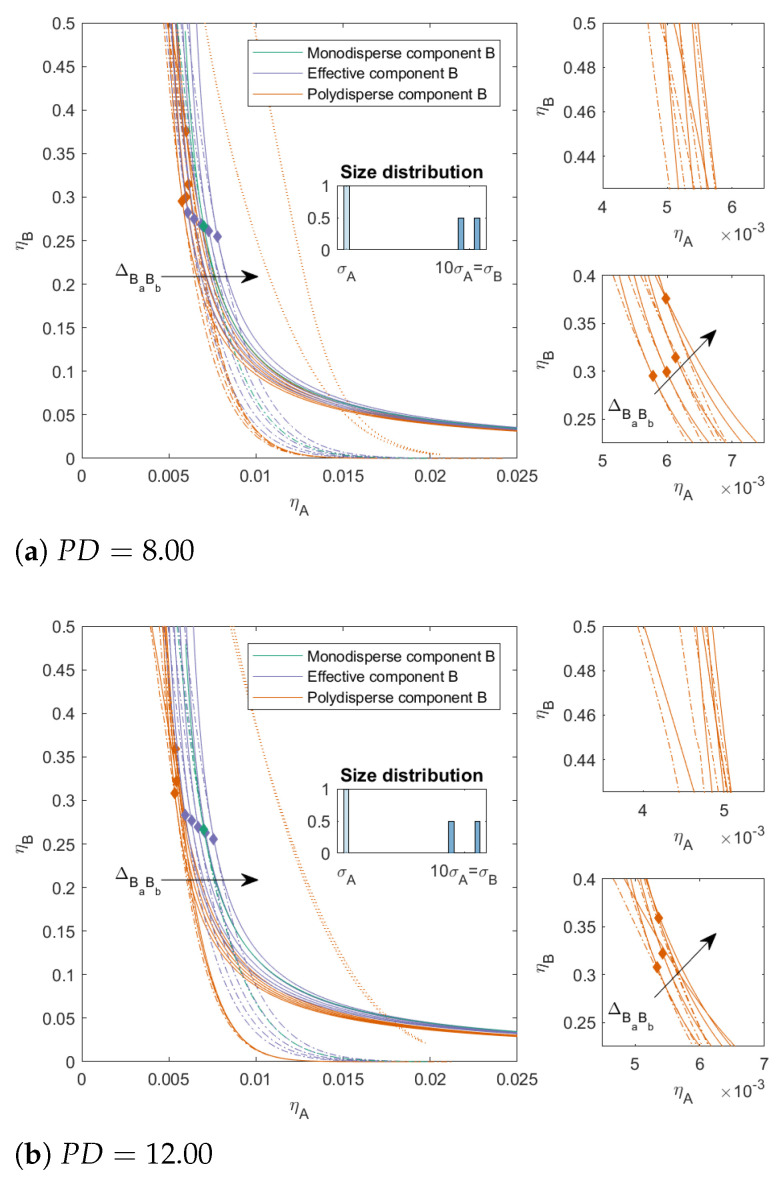
Phase diagram for binary (component *A* and *B*) nonadditive hard sphere mixture with size ratio $q=\sigma_{A}/\sigma_{B}=1/10$ plotted as a function of the partial packing fractions, $\eta_{A}$ and $\eta_{B}$; component *A* is monodisperse, component *B* is polydisperse (PD=8.00 or PD=12.00). The symbols $\sigma_{A}$ and $\sigma_{B}$ refer to the diameters of the species A and B, respectively. The interaction between components *A* and *B* is additive, the interaction between the subcomponents *B* is nonadditive, the nonadditivity parameter $\Delta_{B_{a}B_{b}}$ was varied from −0.1 to 0.1 with a step size of 0.05 (the arrow indicates increasing $\Delta_{B_{a}B_{b}}$). The spinodal (solid line) and binodal (dashed line) meet each other at the plait point (diamond), the three-phase boundary is indicated with a dotted line. The two right-hand smaller figures represent zoomed-in sections of the main figure.

In Figure 4, we increased the PD for component *B* to 8.00 and 12.00, respectively, we kept $\Delta_{AB}=0$, and we varied $\Delta_{B_{a}B_{b}}$ as before. With an increased PD, the two-phase boundary of the polydisperse mixture shifted towards lower packing fractions for all mixtures. The effect of $\Delta_{B_{a}B_{b}}$ on the position of the two-phase boundary became smaller at lower concentration of *B*; however, at higher concentrations of *B*, we see that the two-phase boundary for positive $\Delta_{B_{a}B_{b}}$ bent towards the y-axis, and this effect was more pronounced for higher PD. The polydispersity of *B* also had an effect on the position of the three-phase boundary. With increased PD, the position of the three-phase boundary became less dependent on $\Delta_{B_{a}B_{b}}$ and the difference in the position of the two-phase boundary and the three-phase boundary increased for the mixtures with $\Delta_{B_{a}B_{b}}=0.10$. For the mixtures with PD=12.00, the difference between the three-phase boundary for the mixtures that phase-separated into three phases became negligible. We saw similar trends in the critical points for the more polydisperse mixture as in Figure 3; however, with increased polydispersity and especially increased incompatibility between the subcomponents ($\Delta_{B_{a}B_{b}}>0$), the critical point shifted towards higher concentrations of *B*. For the mixtures with larger $\Delta_{B_{a}B_{b}}$, the critical point could shift to $\eta_{B_{crit}}>0.5$.

**Figure 5:**
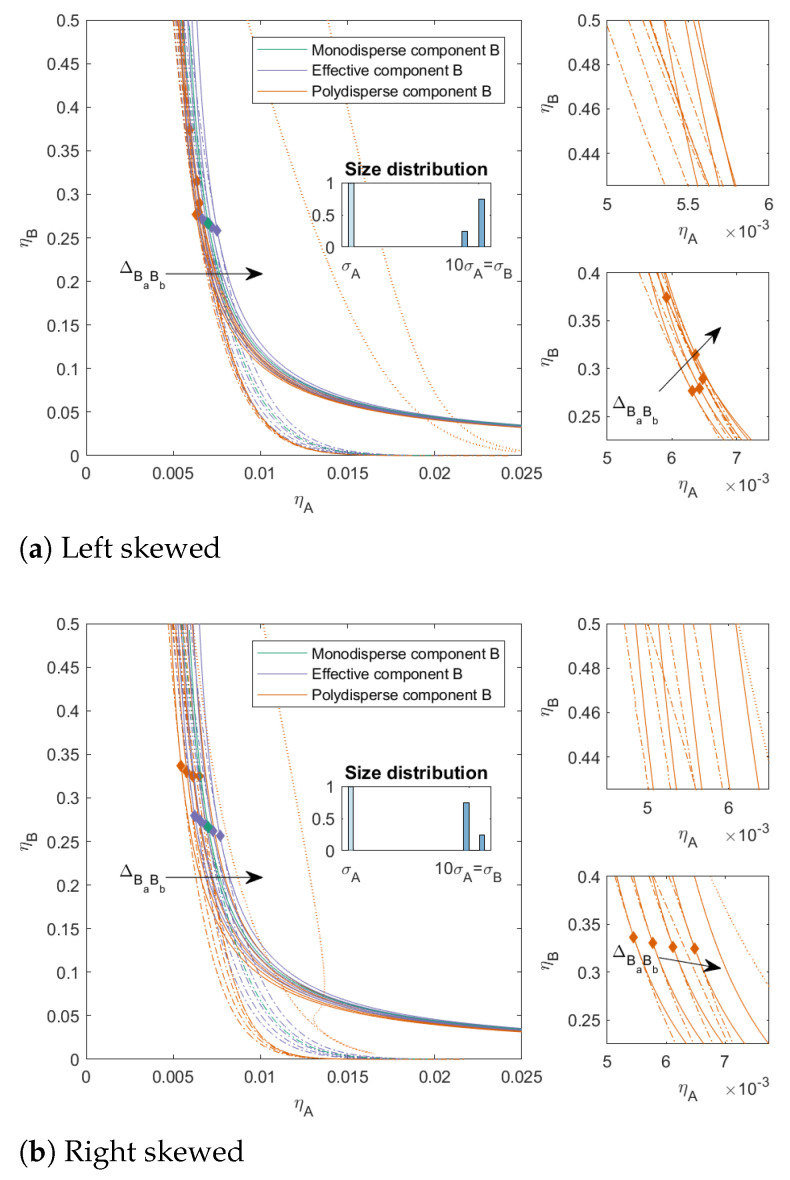
Phase diagram for binary (component *A* and *B*) nonadditive hard sphere mixture with size ratio $q=\sigma_{A}/\sigma_{B}=1/10$ plotted as a function of the partial packing fractions, $\eta_{A}$ and $\eta_{B}$; component *A* is monodisperse, component *B* is polydisperse (PD=6.93). The symbols $\sigma_{A}$ and $\sigma_{B}$ refer to the diameters of the species A and B, respectively. The interaction between components *A* and *B* is additive, the interaction between the subcomponents *B* is nonadditive, the nonadditivity parameter $\Delta_{B_{a}B_{b}}$ was varied from −0.1 to 0.1 with a step size of 0.05 (the arrow indicates increasing $\Delta_{B_{a}B_{b}}$). The spinodal (solid line) and binodal (dashed line) meet each other at the plait point (diamond), the three-phase boundary is indicated with a dotted line. The right-hand smaller figures represent zoomed in versions of the left larger figures.

**Figure 6:**
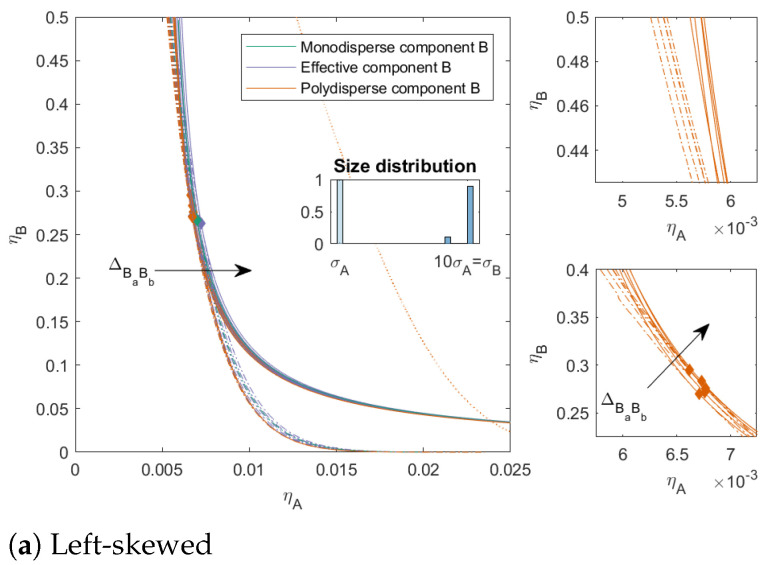

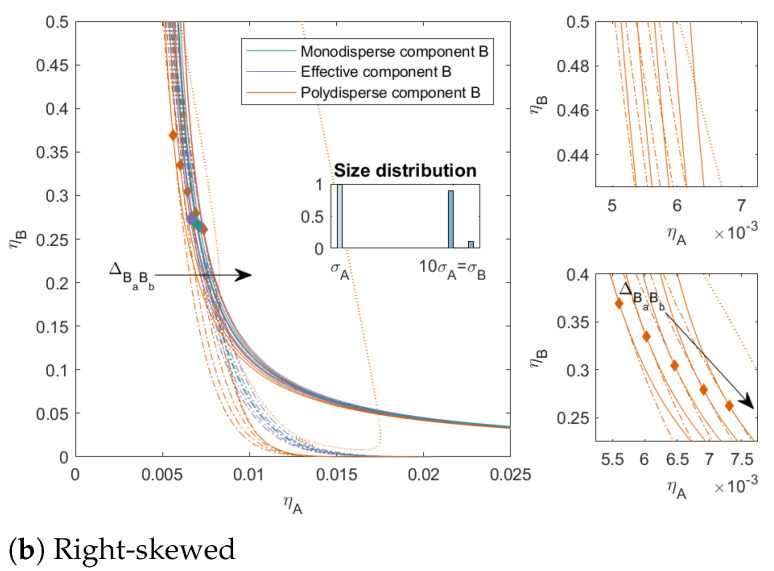
Phase diagram for binary (component *A* and *B*) nonadditive hard sphere mixture with size ratio $q=\sigma_{A}/\sigma_{B}=1/10$ versus the partial packing fractions, $\eta_{A}$ and $\eta_{B}$; component *A* is monodisperse, component *B* is polydisperse (PD=4.80). The symbols $\sigma_{A}$ and $\sigma_{B}$ refer to the diameters of the species A and B, respectively. The interaction between components *A* and *B* is additive, the interaction between the subcomponents *B* nonadditive, the nonadditivity parameter $\Delta_{B_{a}B_{b}}$ was varied from −0.1 to 0.1 with a step size of 0.05 (the arrow indicates increasing $\Delta_{B_{a}B_{b}}$). The spinodal (solid line) and binodal (dashed line) meet each other at the plait point (diamond), the three-phase boundary is indicated with a dotted line. The smaller right-hand figures represent zoomed in versions of the left larger figure.

In Figure 5 and Figure 6, we varied the ratio between the subcomponents of *B*. The ratio between the subcomponents of *B* was 25/75 with a PD=6.93 in Figure 5 (both left and right skewed) and 10/90 with a PD=4.80 in Figure 6 (both left and right skewed). These mixtures can be seen as a model for mixtures that contain some impurities, from a similar material but at different sizes when $\Delta_{B_{a}B_{b}}=0$ or a material that is less compatible with the main component (when $\Delta_{B_{a}B_{b}}>0$) or more compatible with the main component (when $\Delta_{B_{a}B_{b}}<0$). The PD was the same for both the left-skewed and the right-skewed mixtures. For both types of mixtures, we see that the two-phase boundaries were closer to each other for the left-skewed mixtures (large amount of the largest subcomponent) compared to the right-skewed mixtures. Moreover, these left-skewed mixtures also showed a larger bend in the two-phase boundary towards the y-axis for $\Delta_{B_{a}B_{b}}>0$. The mixture in Figure 6a with $\Delta_{B_{a}B_{b}}=0.05$ did not have a three-phase boundary, even though mixtures with these sizes can phase-separate into three phases: the distribution of the subcomponents made these concentrations unattainable in the range of concentrations we focused on.

For the right-skewed mixtures, we see that the three-phase boundary for mixtures with $\Delta_{B_{a}B_{b}}=0.10$ came very close to the two-phase boundary and for mixtures with $\Delta_{B_{a}B_{b}}=0.05$ the three-phase boundary showed a bend back towards lower concentrations of *A* at low concentrations of *B*. This is due to the shape of the three-phase surface and can also be seen on a small level in the mixture $\Delta_{B_{a}B_{b}}=0.10$ in Figure 3.

Furthermore, [27] found the possibility of three-phase separation for polydisperse components. According to them, the transition between the two-phase and three-phase region proceeds via a second critical point. This second critical point is polydispersity induced.

### 3.3. Mixtures with Nonadditivity between Subcomponents of *B* ($\Delta_{B_{a}B_{b}}$), and between *A* and *B* ($\Delta_{AB}$)

**Figure 7:**
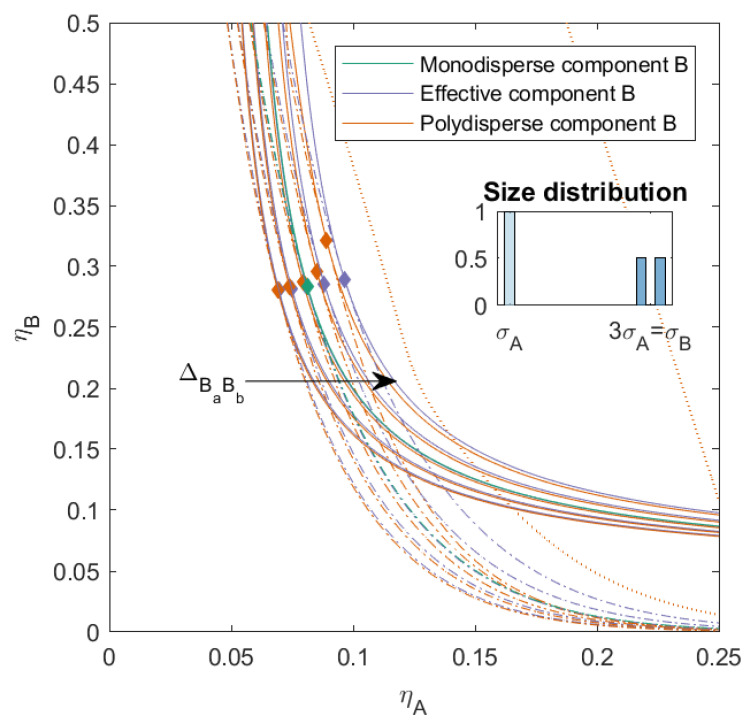
Phase diagram for binary (component *A* and *B*) nonadditive hard sphere mixture with size ratio $q=\sigma_{A}/\sigma_{B}=1/3$ plotted as a function of the partial packing fractions, $\eta_{A}$ and $\eta_{B}$; component *A* is monodisperse, component *B* is polydisperse (PD=4.00). The symbols $\sigma_{A}$ and $\sigma_{B}$ refer to the diameters of the species A and B, respectively. The interaction between components *A* and *B* is nonadditive with a nonadditivity parameter $\Delta_{AB}=0.075$, the interaction between the subcomponents *B* is nonadditive, the nonadditivity parameter $\Delta_{B_{a}B_{b}}$ was varied from −0.1 to 0.1 with a step size of 0.05 (the arrow indicates increasing $\Delta_{B_{a}B_{b}}$). The spinodal (solid line) and binodal (dashed line) meet each other at the plait point (diamond), the three-phase boundary is indicated with a dotted line.

In Figure 7, we plotted the phase diagram for mixtures with varying $\Delta_{B_{a}B_{b}}$, with a size ratio between component *A* and *B* of q=1/3, and a nonadditive interaction between *A* and *B*$\Delta_{AB}=0.075$. This was in fact a combination of the cases in Section 3.1 and Section 3.2 at a lower size ratio between *A* and *B*. The polydispersity of *B* was 4.00 (for mixtures with more variety in PD and $\Delta_{AB}$, we refer the reader to the Supplementary Materials). The phase diagram of these mixtures showed a lot of similarities with the phase diagram of the mixtures in Figure 3, though at different $\eta_{A}$ due to the different size ratio. Since the mixtures in Figure 3 had the same PD, we concluded that the three-phase boundary position and shape was largely dependent on the interaction between the subcomponents of *B*. The interaction between the subcomponents was determined by both the PD and the nonadditivity parameter $\Delta_{B_{a}B_{b}}$.

### 3.4. Fractionation

When a parent mixture demixes into two or more phases, each component (and also their subcomponents) in the mixture finds its preferential phase in order to minimize the Helmholtz free energy of the system. This leads each phase to be enriched in one of the components, whilst being depleted by the other component(s). The other components are then present only at low volume fractions. We investigated the phase separation for the mixtures in Section 3.2 for a specific parent mixture ($\eta_{A_{parent}}=0.010,\eta_{B_{parent}}=0.200$) in terms of the volume fraction of both components in each phase, the degree of polydispersity of component *B*, the average size of component *B* in the child phases compared to the average size of component *B* in the parent phase and the volume fraction of the phases (α), see Table 1 for the mixtures from Figure 3 (mixtures with PD=4.00). For other mixtures, we refer the reader to the Supplementary Materials. The composition histograms for each phase are given in Table 2 for the same mixture; for other mixtures we refer the reader to the Supplementary Materials. Since at this parent concentration, the mixture with nonadditivity parameter $\Delta_{B_{a}B_{b}}=0.10$ separates into three phases, we also calculated the child phases for mixtures with between $\Delta_{B_{a}B_{b}}=0.10$ and $\Delta_{B_{a}B_{b}}=0.05$ to investigate the behavior of the subcomponents *B* depending on the nonadditivity.

For all mixtures, the top phase, which was also the largest phase in volume, was enriched in component *A*. The volume fraction of the top phase was dependent on the nonadditive interaction between the subcomponents of *B*. It increased with both more compatibility between the subcomponents as well as less compatibility, with a minimum volume fraction at $\Delta_{B_{a}B_{b}}=0.05$. We also found this dependence in volume fraction on the nonadditivity parameter $\Delta_{B_{a}B_{b}}$ for the other mixtures; however, the minimum volume fraction was at different $\Delta_{B_{a}B_{b}}$ depending on the sizes and the ratio of the subcomponents *a* and *b* of *B*. For the mixtures ($\Delta_{B_{a}B_{b}}>0.075$) that phase-separated into three phases at this parent mixture concentration, we concluded that it was mostly the bottom phase that demixed into an additional phase (the middle phase). The bottom phase was enriched in the largest subcomponent of *B*, while the top phase (and middle phase to a lesser extent) was enriched in the smaller subcomponent of *B*. We also saw this behavior for the other mixtures with a different composition of *B*.

The fractionation of the subcomponents of *B* was dependent on the nonadditivity parameter $\Delta_{B_{a}B_{b}}$. When $\Delta_{B_{a}B_{b}}<0$, the subcomponents *a* and *b* were more compatible with each other and this led to less fractionation, as can be seen in Table 2, while on the other hand, when $\Delta_{B_{a}B_{b}}>0$, the subcomponents were less compatible with each other and more fractionation occurred, even leading to additional phase separation at higher $\Delta_{B_{a}B_{b}}$. This is something we also saw for the other mixtures (see Supplementary Material).

## 4. Conclusions

We found that when the compatibility between component *A* and *B* was decreased, the phase diagram (the critical point, phase boundary, and spinodal) shifted towards lower volume fractions. This was in line with the literature on the phase behavior of NAHS binary monodisperse mixtures. The interaction between *A* and *B* was driven by the size ratio (*q*) between *A* and *B* and the nonadditivity parameter $\Delta_{AB}$.

When the compatibility between the subcomponents of the polydisperse component *B* was altered, the phase diagram changed more drastically. When the compatibility between the subcomponents was decreased, the mixture could demix into three phases, each enriched in one of the (sub)components of the parent mixture. The shape and position of the three-phase boundary was mainly dependent on the interactions between the subcomponents of *B*. This meant that it was dependent on the nonadditivity parameter ($\Delta_{B_{a}B_{b}}$) as well as the size ratios and distribution of the subcomponents (the degree of polydispersity PD). Moreover, depending on the size ratios and distribution of the subcomponents, we also saw that the binodal and spinodal bent towards the y-axis for higher volume fractions of *B* when $\Delta_{B_{a}B_{b}}$ increased. For the mixtures with a more pronounced bend in the phase boundary and spinodal, we found that the critical point shifted to volume fractions $\eta_{B_{crit}}>0.5$. This behavior was driven to a large extent by the nonadditivity parameter ($\Delta_{B_{a}B_{b}}$) as well as the size ratios and distribution of the subcomponents (the degree of polydispersity PD), and to a lesser extent, by the interaction between *A* and *B*. When the compatibility between the subcomponents was increased, the mixture demixed at slightly lower packing fractions compared to the HS mixture. The fractionation of the polydisperse subcomponents of *B* was also dependent on the nonadditivity parameter $\Delta_{B_{a}B_{b}}$. Less fractionation occurred when $\Delta_{B_{a}B_{b}}<0$ and more fractionation occurred when $\Delta_{B_{a}B_{b}}>0$. At higher $\Delta_{B_{a}B_{b}}$, this could even lead to additional phase separation, creating a third phase.

The virial coefficient approach for polydisperse mixtures allows for the prediction of the phase behavior of polydisperse or impure binary mixtures. Not only does it allow for plotting the phase diagram, it also allows for the calculation of the composition and fractionation of each component in each phase.

## Figures and Tables

**Table 1:** Critical points are given for the different binary mixtures, depending on the nonadditivity of component *B* (see also Figure 3), phase-separated concentrations, and volume fraction α of the different mixtures for a specific parent concentration ($\eta_{A_{parent}}=0.010,\eta_{B_{parent}}=0.200$), depending on the nonadditivity of component *B*. See also Table 2 for a distribution of component *B* in each phase.

$\Delta_{B_{a}B_{b}}$	$\eta_{\mathrm{crit}}$	Top Phase	Middle Phase	Bottom Phase
0.100	(0.007, 0.323)	η (0.011, 0.074)	η (0.006, 0.596)	η (0.004,0.953)
		PD: 3.35, Size: 0.97, α: 0.817	PD: 3.84, Size: 0.99, α: 0.097	PD: 2.62, Size: 1.03, α: 0.087
0.0875	(0.007, 0.303)	η (0.011, 0.064)	η (0.005, 0.733)	η (0.004, 0.813)
		PD: 3.39, Size: 0.98, α: 0.804	PD: 3.94, Size: 1.01, α: 0.138	PD: 3.65, Size: 1.02, α: 0.058
0.075	(0.007, 0.294)	η (0.011, 0.057)		η (0.005, 0.767)
		PD: 3.42, Size: 0.98, α: 0.799		PD: 3.91, Size: 1.01, α: 0.201
0.05	(0.007, 0.285)	η (0.011, 0.047)		η (0.004, 0.800)
		PD: 3.44, Size: 0.98, α: 0.797		PD: 3.94, Size: 1.01, α: 0.203
0	(0.007, 0.280)	η (0.011, 0.034)		η (0.004, 0.881)
		PD: 3.45, Size: 0.98, α: 0.804		PD: 3.97, Size: 1.00, α: 0.196
−0.05	(0.006, 0.281)	η (0.011, 0.026)		η (0.004, 0.966)
		PD: 3.45, Size: 0.98, α: 0.815		PD: 3.98, Size: 1.00, α: 0.185
−0.1	(0.006, 0.285)	η (0.011, 0.020)		η (0.003, 1.051)
		PD: 3.45, Size: 0.98, α: 0.825		PD: 3.99, Size: 1.00, α: 0.175

**Table 2:** Phase separation of different mixtures and fractionation of component *B* for a specific parent distribution ($\eta_{A_{parent}}=0.010,\eta_{B_{parent}}=0.200$), depending on the nonadditivity of component *B*, see also Figure 3.

$\Delta_{B_{a}B_{b}}$	Parent Dist.	Top Phase	Middle Phase	Bottom Phase
0.1	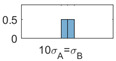	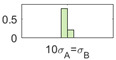	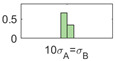	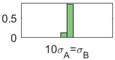
0.0875	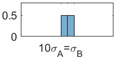	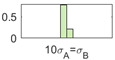	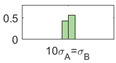	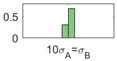
0.075	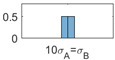	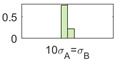		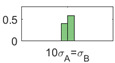
0.05	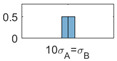	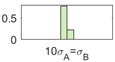		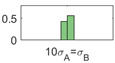
0	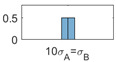	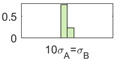		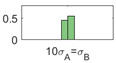
−0.05	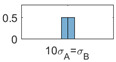	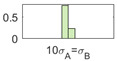		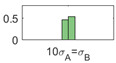
−0.1	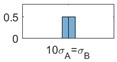	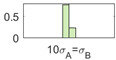		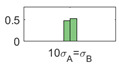

## Data Availability

Not applicable.

## Supplementary Materials

The following supporting information can be downloaded at: https://www.mdpi.com/article/10.3390/molecules27196354/s1.
