# Supplementary material for: Towards Predicting Partitioning of Enzymes between Macromolecular Phases: Effects of Polydispersity on the Phase Behavior of Nonadditive Hard Spheres in Solution
Source: Molecules. 2022 Sep 26;27(19):6354. doi: 10.3390/molecules27196354 (PMC9573134; doi:10.3390/molecules27196354)
Supplement: Supplementary file 1 [file molecules-27-06354-s001.zip › molecules-1812471-supplementary.pdf]

Supplementary Materials

Luka Sturtewagen[1] and Erik van der Linden[1]

Laboratory of Physics and Physical Chemistry of Foods,

Wageningen University, Bornse Weilanden 9, 6708 WG Wageningen,

The Netherlands

I. FIGURES

A. Variation in Δ_{AB} between component A and B

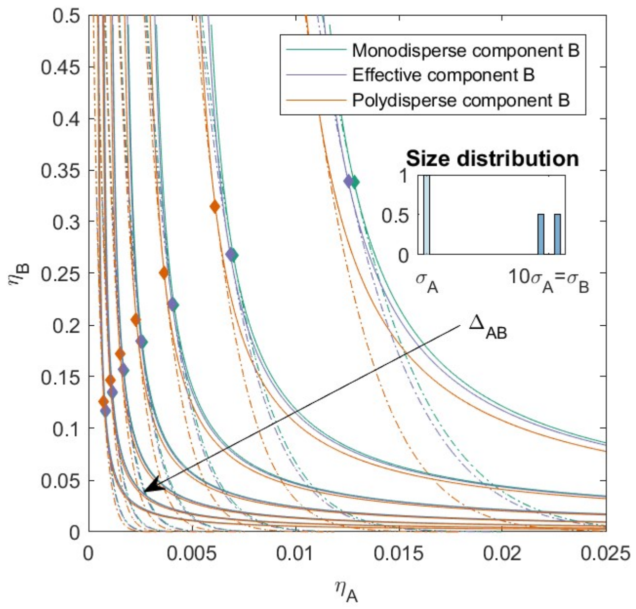

(a) $PD = 8.00$

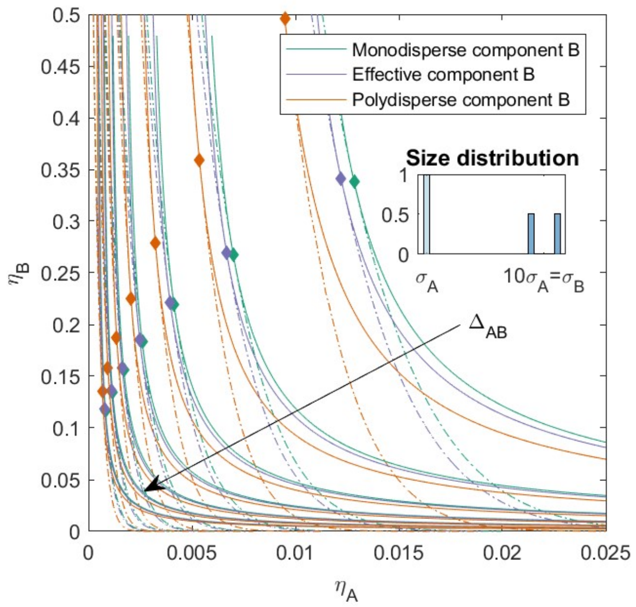

(b) $PD = 12.00$

FIG. S 1. Phase diagram for binary (component A and B) non-additive hard sphere mixture with size ratio $q = \sigma_A/\sigma_B = 1/10$, component A is monodisperse, component B is polydisperse, plotted as a function of the partial packing fractions, η_A and η_B. The interaction between components A and B is non-additive, the non-additivity parameter Δ_{AB} was varied from -0.1 to 0.5 with a step size of 0.1 (the arrow indicates increasing Δ_{AB}). The interaction between the sub-components B is additive. The spinodal (solid line) and binodal (dashed line) meet each other at the critical point (diamond)

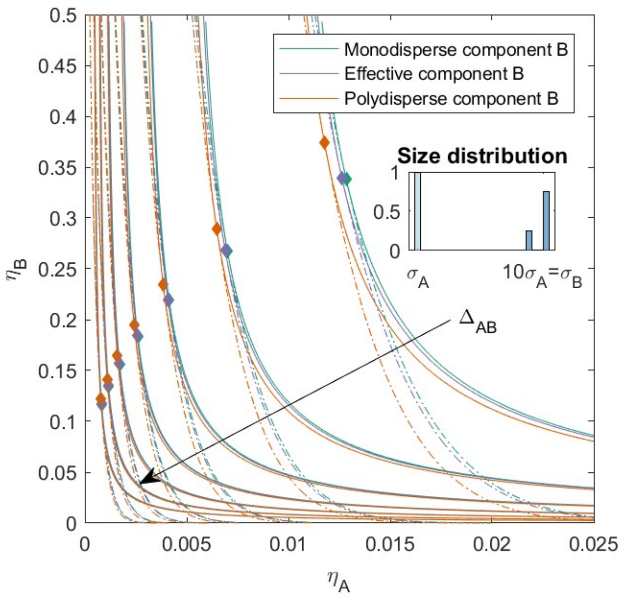

(a) Left skewed

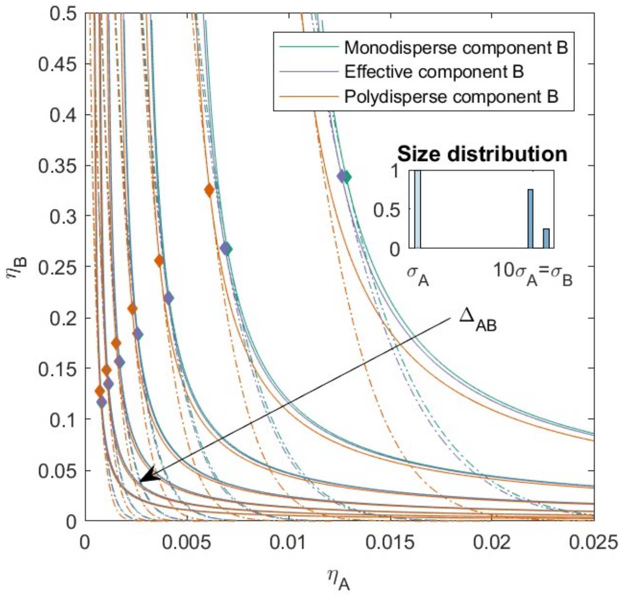

(b) Right skewed

FIG. S 2. Phase diagram for binary (component A and B) non-additive hard sphere mixture with size ratio $q = \sigma_A/\sigma_B = 1/10$, component A is monodisperse, component B is polydisperse ($PD = 6.93$), plotted as a function of the partial packing fractions, η_A and η_B. The interaction between components A and B is non-additive, the non-additivity parameter Δ_{AB} was varied from -0.1 to 0.5 with a step size of 0.1 (the arrow indicates increasing Δ_{AB}). The interaction between the sub-components B is additive. The spinodal (solid line) and binodal (dashed line) meet each other at the critical point (diamond)

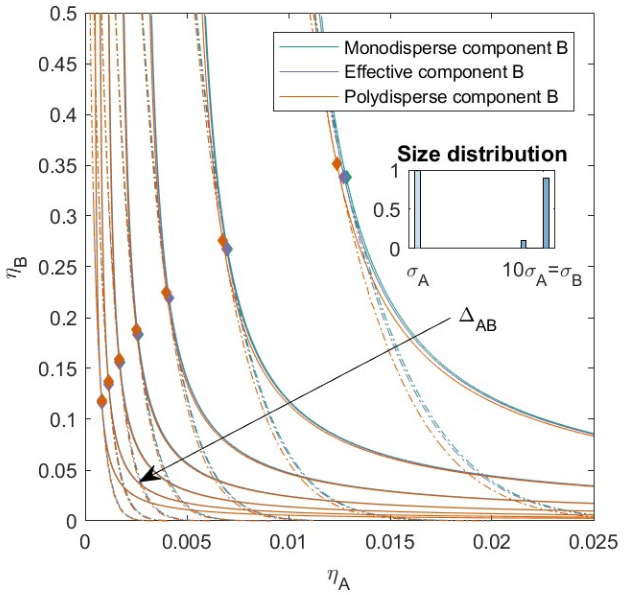

(a) Left skewed

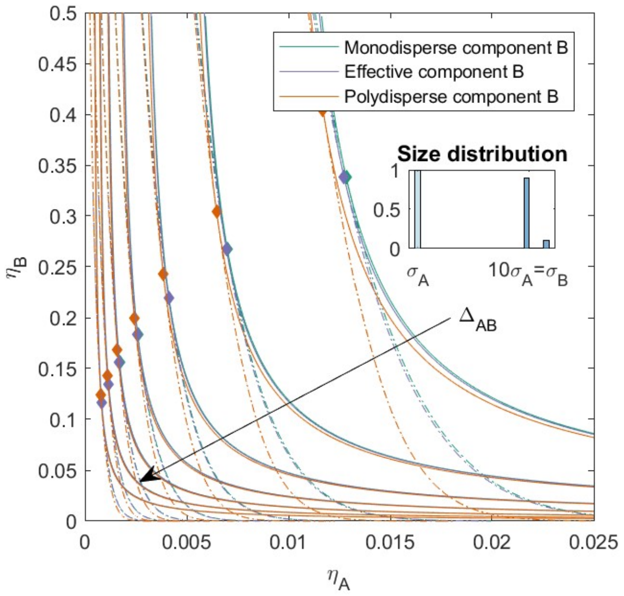

(b) Right skewed

FIG. S 3. Phase diagram for binary (component A and B) non-additive hard sphere mixture with size ratio $q = \sigma_A/\sigma_B = 1/10$, component A is monodisperse, component B is polydisperse ($PD = 4.80$), plotted as a function of the partial packing fractions, η_A and η_B. The interaction between components A and B is non-additive, the non-additivity parameter Δ_{AB} was varied from -0.1 to 0.5 with a step size of 0.1 (the arrow indicates increasing Δ_{AB}). The interaction between the sub-components B is additive. The spinodal (solid line) and binodal (dashed line) meet each other at the critical point (diamond)

B. Variation in $\Delta_{B_aB_b}$ between sub-components B at lower size ratio (q) between component A and B for different Δ_{AB}

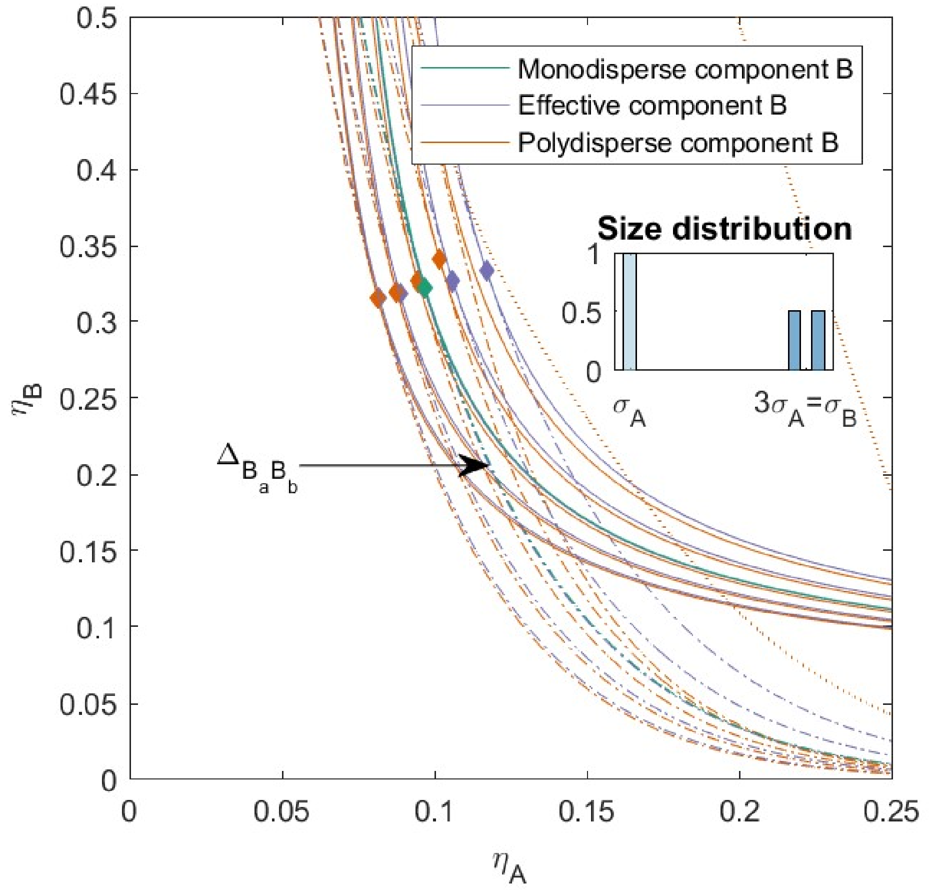

FIG. S 4. Phase diagram for binary (component A and B) non-additive hard sphere mixture with size ratio $q = \sigma_A/\sigma_B = 1/3$, component A is monodisperse, component B is polydisperse ($PD = 4.00$), plotted as a function of the partial packing fractions, η_A and η_B. The interaction between components A and B is non-additive with a non-additivity parameter $\Delta_{AB} = 0.05$, the interaction between the sub-components B is non-additive, the non-additivity parameter $\Delta_{B_aB_b}$ was varied from -0.1 to 0.1 with a step size of 0.05 (the arrow indicates increasing $\Delta_{B_aB_b}$). The spinodal (solid line) and binodal (dashed line) meet each other at the plait point (diamond), the three phase boundary is indicated with a dotted line

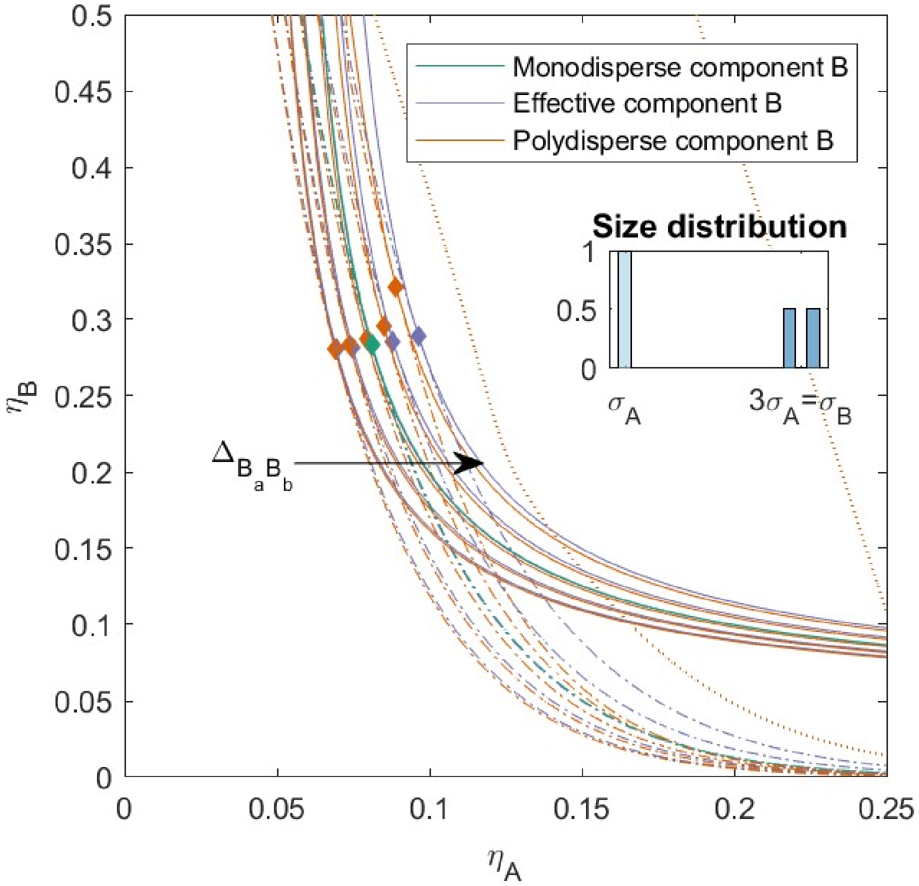

FIG. S 5. Phase diagram for binary (component A and B) non-additive hard sphere mixture with size ratio $q = \sigma_A/\sigma_B = 1/3$, component A is monodisperse, component B is polydisperse ($PD = 4.00$), plotted as a function of the partial packing fractions, η_A and η_B. The interaction between components A and B is non-additive with a non-additivity parameter $\Delta_{AB} = 0.075$, the interaction between the sub-components B is non-additive, the non-additivity parameter $\Delta_{B_aB_b}$ was varied from -0.1 to 0.1 with a step size of 0.05 (the arrow indicates increasing $\Delta_{B_aB_b}$). The spinodal (solid line) and binodal (dashed line) meet each other at the plait point (diamond), the three phase boundary is indicated with a dotted line

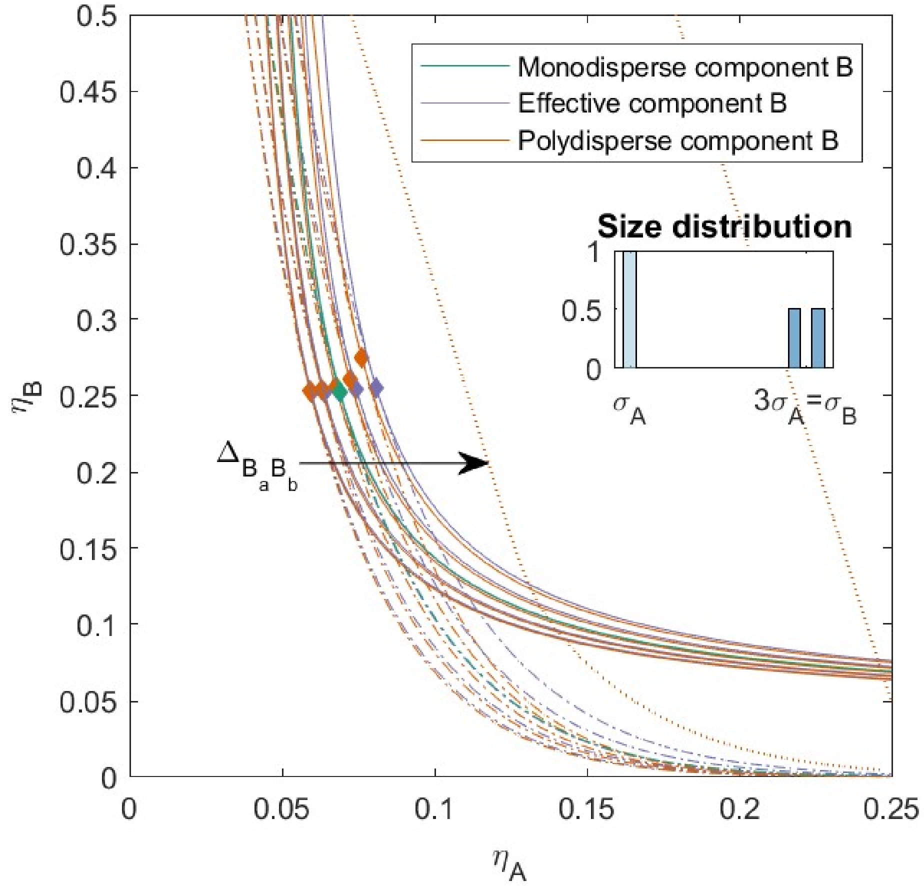

FIG. S 6. Phase diagram for binary (component A and B) non-additive hard sphere mixture with size ratio $q = \sigma_A/\sigma_B = 1/3$, component A is monodisperse, component B is polydisperse ($PD = 4.00$), plotted as a function of the partial packing fractions, η_A and η_B. The interaction between components A and B is non-additive with a non-additivity parameter $\Delta_{AB} = 0.10$, the interaction between the sub-components B is non-additive, the non-additivity parameter $\Delta_{B_aB_b}$ was varied from -0.1 to 0.1 with a step size of 0.05 (the arrow indicates increasing $\Delta_{B_aB_b}$). The spinodal (solid line) and binodal (dashed line) meet each other at the plait point (diamond), the three phase boundary is indicated with a dotted line

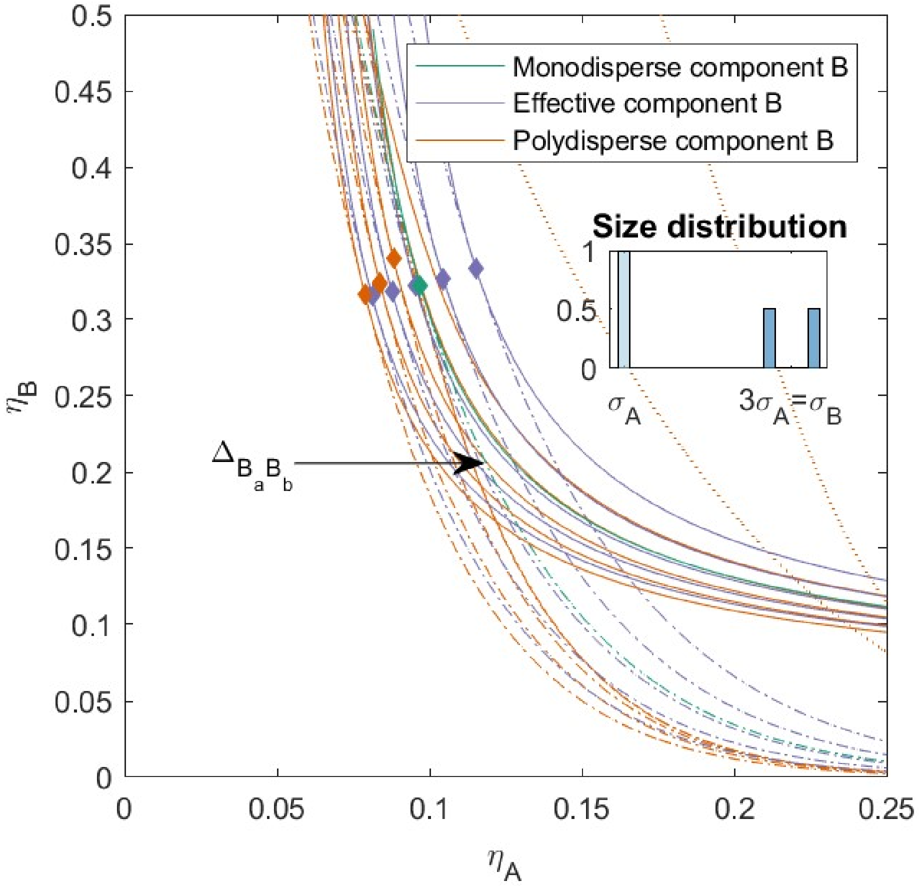

FIG. S 7. Phase diagram for binary (component A and B) non-additive hard sphere mixture with size ratio $q = \sigma_A/\sigma_B = 1/3$, component A is monodisperse, component B is polydisperse ($PD = 8.00$), plotted as a function of the partial packing fractions, η_A and η_B. The interaction between components A and B is non-additive with a non-additivity parameter $\Delta_{AB} = 0.05$, the interaction between the sub-components B is non-additive, the non-additivity parameter $\Delta_{B_aB_b}$ was varied from -0.1 to 0.1 with a step size of 0.05 (the arrow indicates increasing $\Delta_{B_aB_b}$). The spinodal (solid line) and binodal (dashed line) meet each other at the plait point (diamond), the three phase boundary is indicated with a dotted line

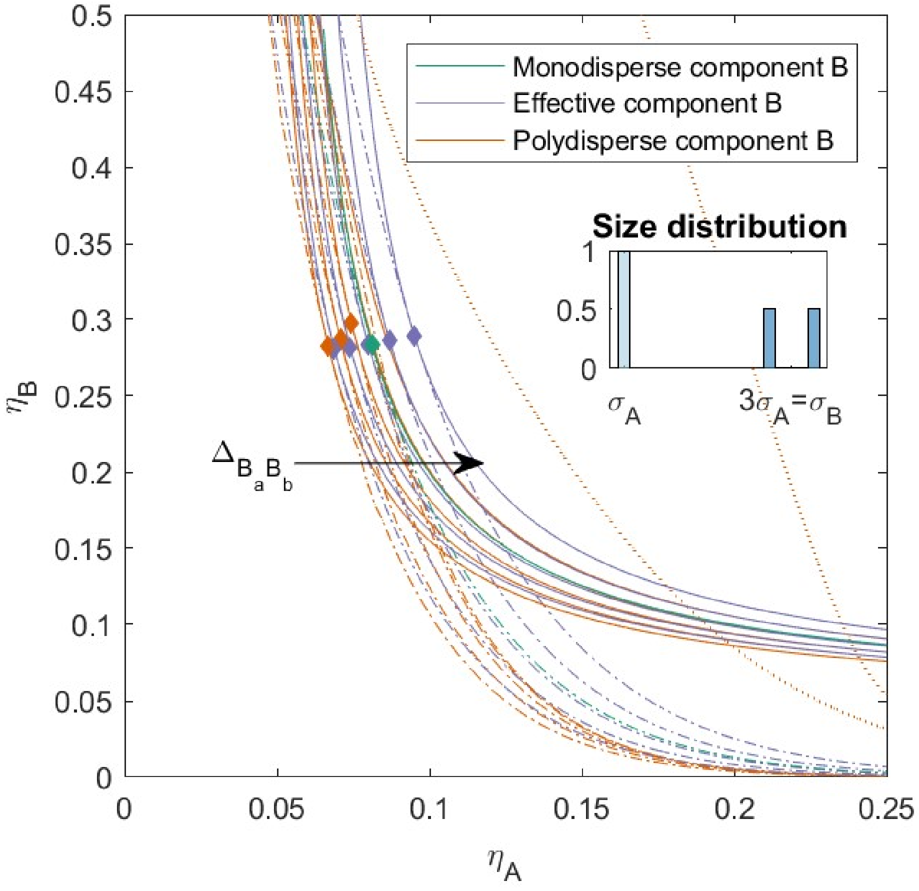

FIG. S 8. Phase diagram for binary (component A and B) non-additive hard sphere mixture with size ratio $q = \sigma_A/\sigma_B = 1/3$, component A is monodisperse, component B is polydisperse ($PD = 8.00$), plotted as a function of the partial packing fractions, η_A and η_B. The interaction between components A and B is non-additive with a non-additivity parameter $\Delta_{AB} = 0.075$, the interaction between the sub-components B is non-additive, the non-additivity parameter $\Delta_{B_aB_b}$ was varied from -0.1 to 0.1 with a step size of 0.05 (the arrow indicates increasing $\Delta_{B_aB_b}$). The spinodal (solid line) and binodal (dashed line) meet each other at the plait point (diamond), the three phase boundary is indicated with a dotted line

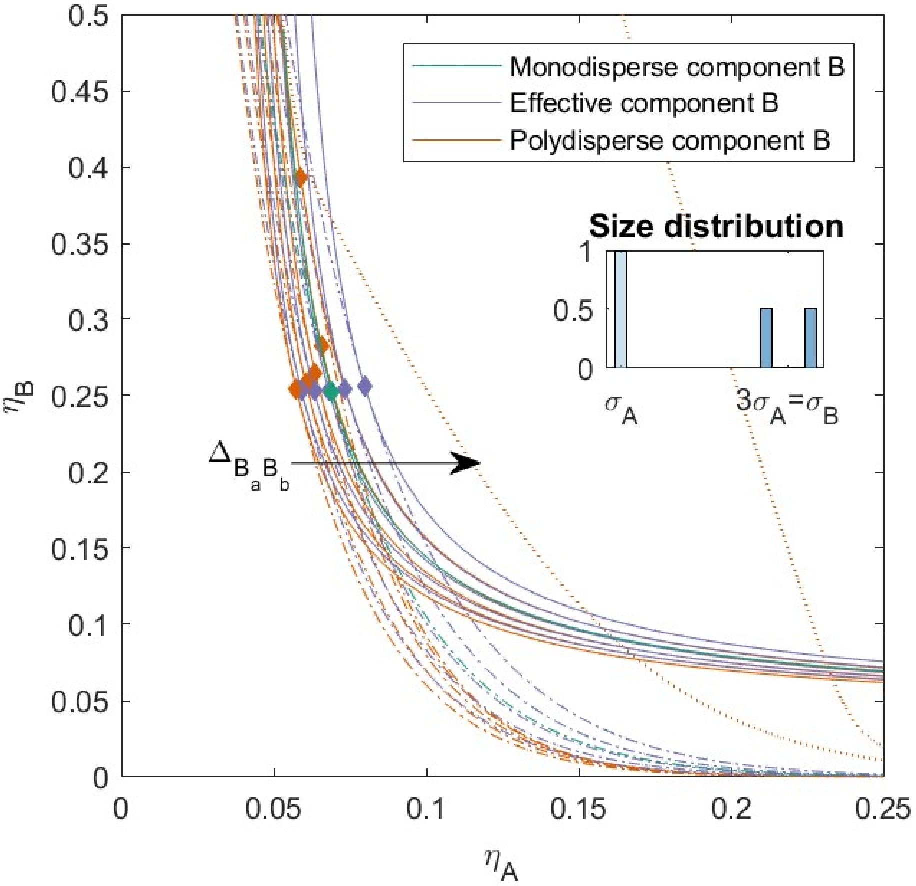

FIG. S 9. Phase diagram for binary (component A and B) non-additive hard sphere mixture with size ratio $q = \sigma_A/\sigma_B = 1/3$, component A is monodisperse, component B is polydisperse ($PD = 8.00$), plotted as a function of the partial packing fractions, η_A and η_B. The interaction between components A and B is non-additive with a non-additivity parameter $\Delta_{AB} = 0.1$, the interaction between the sub-components B is non-additive, the non-additivity parameter $\Delta_{B_aB_b}$ was varied from -0.1 to 0.1 with a step size of 0.05 (the arrow indicates increasing $\Delta_{B_aB_b}$). The spinodal (solid line) and binodal (dashed line) meet each other at the plait point (diamond), the three phase boundary is indicated with a dotted line

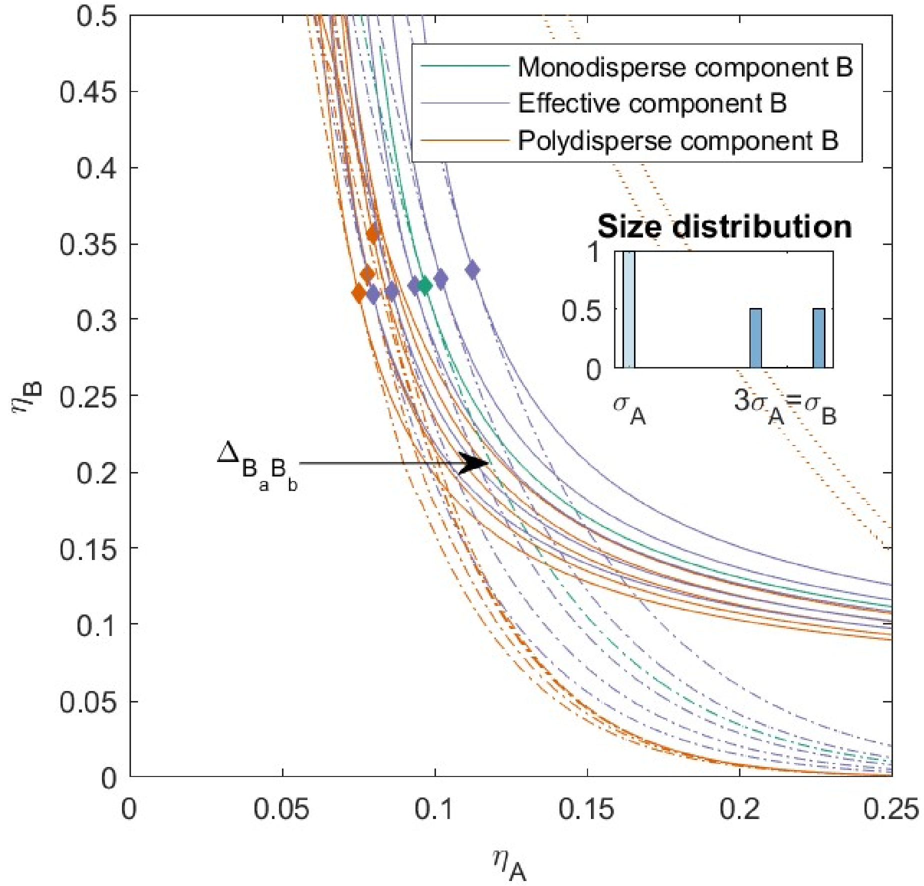

FIG. S 10. Phase diagram for binary (component A and B) non-additive hard sphere mixture with size ratio $q = \sigma_A/\sigma_B = 1/3$, component A is monodisperse, component B is polydisperse ($PD = 12.00$), plotted as a function of the partial packing fractions, η_A and η_B. The interaction between components A and B is non-additive with a non-additivity parameter $\Delta_{AB} = 0.05$, the interaction between the sub-components B is non-additive, the non-additivity parameter $\Delta_{B_aB_b}$ was varied from -0.1 to 0.1 with a step size of 0.05 (the arrow indicates increasing $\Delta_{B_aB_b}$). The spinodal (solid line) and binodal (dashed line) meet each other at the plait point (diamond), the three phase boundary is indicated with a dotted line

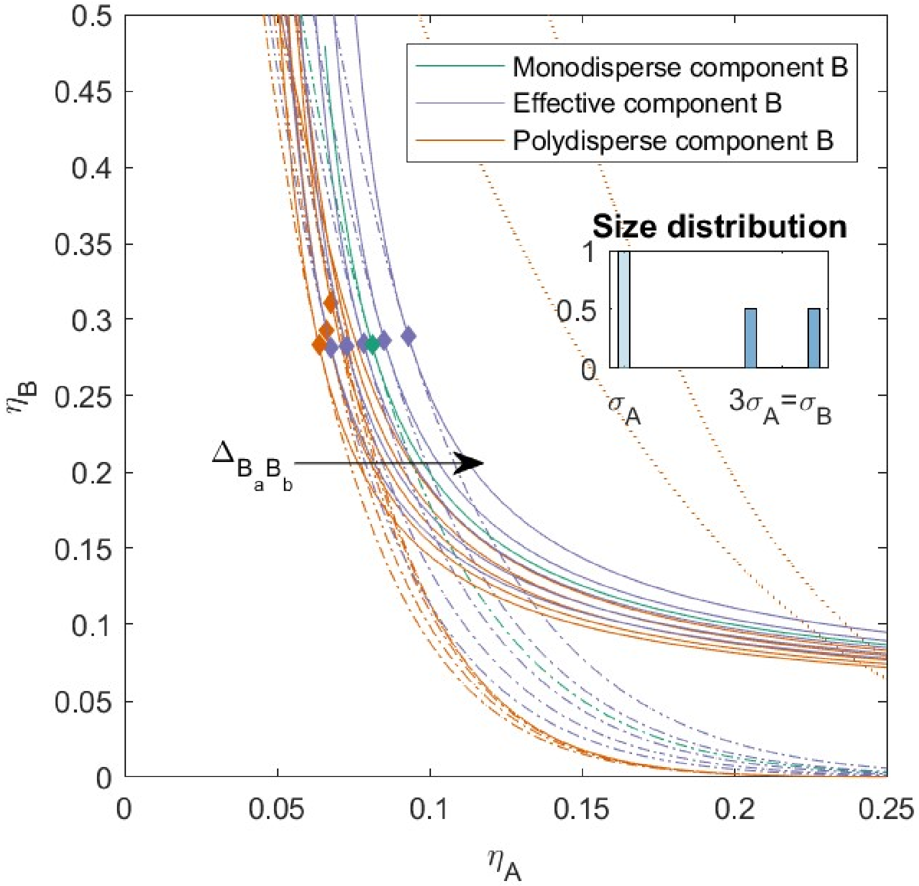

FIG. S 11. Phase diagram for binary (component A and B) non-additive hard sphere mixture with size ratio $q = \sigma_A/\sigma_B = 1/3$, component A is monodisperse, component B is polydisperse ($PD = 12.00$), plotted as a function of the partial packing fractions, η_A and η_B. The interaction between components A and B is non-additive with a non-additivity parameter $\Delta_{AB} = 0.075$, the interaction between the sub-components B is non-additive, the non-additivity parameter $\Delta_{B_aB_b}$ was varied from -0.1 to 0.1 with a step size of 0.05 (the arrow indicates increasing $\Delta_{B_aB_b}$). The spinodal (solid line) and binodal (dashed line) meet each other at the plait point (diamond), the three phase boundary is indicated with a dotted line

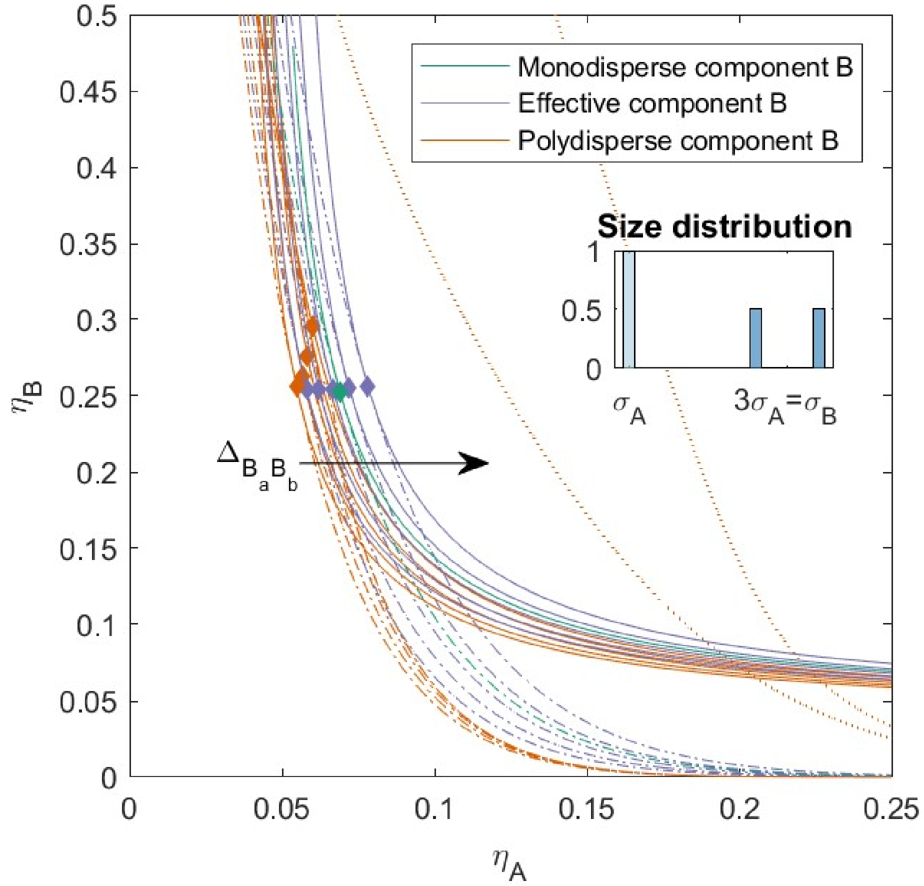

FIG. S 12. Phase diagram for binary (component A and B) non-additive hard sphere mixture with size ratio $q = \sigma_A/\sigma_B = 1/3$, component A is monodisperse, component B is polydisperse ($PD = 12.00$), plotted as a function of the partial packing fractions, η_A and η_B. The interaction between components A and B is non-additive with a non-additivity parameter $\Delta_{AB} = 0.1$, the interaction between the sub-components B is non-additive, the non-additivity parameter $\Delta_{B_aB_b}$ was varied from -0.1 to 0.1 with a step size of 0.05 (the arrow indicates increasing $\Delta_{B_aB_b}$). The spinodal (solid line) and binodal (dashed line) meet each other at the plait point (diamond), the three phase boundary is indicated with a dotted line

II. FRACTIONATION

Table S I. Critical points for the different binary mixtures depending on the non-additivity of component B, and phase separated concentrations and fractionation of the different mixtures for specific parent concentration ($\eta_{A_{parent}} = 0.010, \eta_{B_{parent}} = 0.200$), depending on the non-additivity of component B, $PD = 8.00$

$\Delta_{B_aB_b}$	η_{crit}	**Top phase**	**Bottom phase**
0.100	-	η (0.011, 0.092)<br>PD: 4.65, Size: 0.93,<br>α : 0.903	η (0.003, 1.206)<br>PD: 3.10, Size: 1.07,<br>α : 0.097
	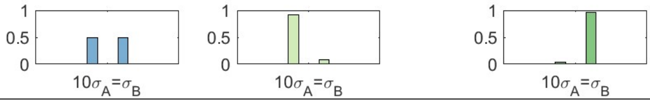		
0.05	(0.006, 0.376)	η (0.011, 0.070)<br>PD: 4.77, Size: 0.93,<br>α : 0.865	η (0.003, 1.032)<br>PD: 6.63, Size: 1.04,<br>α : 0.135
	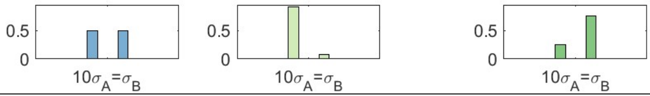		
0	(0.006, 0.314)	η (0.011, 0.045)<br>PD: 4.86, Size: 0.93,<br>α : 0.837	η (0.003, 0.994)<br>PD: 7.58, Size: 1.02,<br>α : 0.163
	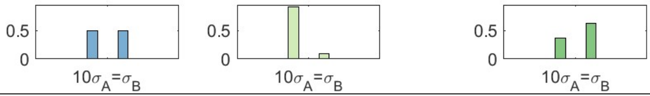		
-0.05	(0.006, 0.300)	η (0.011, 0.033)<br>PD: 4.86, Size: 0.93,<br>α : 0.835	η (0.003, 1.043)<br>PD: 7.78, Size: 1.01,<br>α : 0.165
	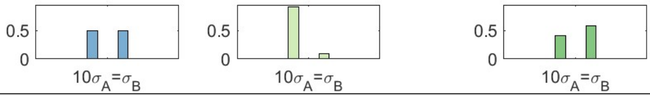		
-0.1	(0.006, 0.295)	η (0.011, 0.025)<br>PD: 4.84, Size: 0.93,<br>α : 0.839	η (0.003, 1.110)<br>PD: 7.86, Size: 1.01,<br>α : 0.161
	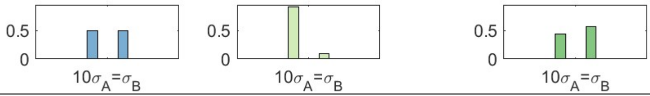		

Table S II. Critical points for the different binary mixtures depending on the non-additivity of component B, and phase separated concentrations and volume fraction α of the different mixtures for specific parent concentration ($\eta_{A_{parent}} = 0.010, \eta_{B_{parent}} = 0.200$), depending on the non-additivity of component B, $PD = 12.00$

$\Delta_{B_aB_b}$	η_{crit}	**Top phase**	**Bottom phase**
0.05	-	η (0.011, 0.071) PD: 4.80, Size: 0.89, α : 0.897	η (0.002, 1.319) PD: 6.08, Size: 1.10, α : 0.103
	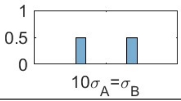	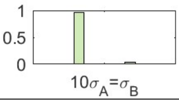	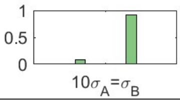
0	(0.005, 0.359)	η (0.011, 0.056) PD: 4.84, Size: 0.89, α : 0.875	η (0.003, 1.210) PD: 9.58, Size: 1.06, α : 0.125
	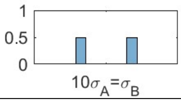	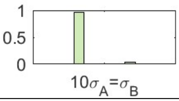	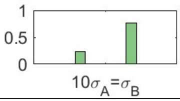
-0.05	(0.005, 0.322)	η (0.011, 0.041) PD: 4.80, Size: 0.89, α : 0.861	η (0.003, 1.184) PD: 10.90, Size: 1.04, α : 0.139
	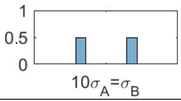	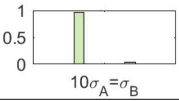	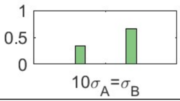
-0.1	(0.005, 0.308)	η (0.011, 0.031) PD: 4.73, Size: 0.89, α : 0.857	η (0.003, 1.214) PD: 11.37, Size: 1.03, α : 0.143
	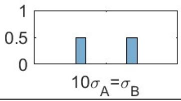	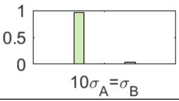	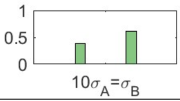

Table S III. Critical points for the different binary mixtures depending on the non-additivity of component B, and phase separated concentrations and volume fraction α of the different mixtures for specific parent concentration ($\eta_{A_{parent}} = 0.010, \eta_{B_{parent}} = 0.200$), depending on the non-additivity of component B, $PD = 6.93$

$\Delta_{B_aB_b}$	η_{crit}	**Top phase**	**Bottom phase**
0.100	(0.006, 0.374)	η (0.011, 0.055) PD: 7.41, Size: 0.92, α : 0.850	η (0.003, 1.024) PD: 2.55, Size: 1.04, α : 0.159
	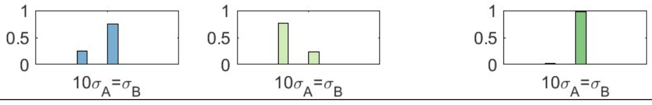		
0.05	(0.006, 0.314)	η (0.011, 0.048) PD: 7.62, Size: 0.92, α : 0.837	η (0.004, 0.978) PD: 4.28, Size: 1.03, α : 0.163
	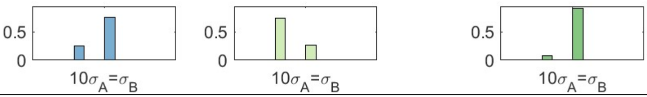		
0	(0.006, 0.290)	η (0.011, 0.037) PD: 7.92, Size: 0.93, α : 0.822	η (0.004, 0.951) PD: 5.57, Size: 1.02, α : 0.178
	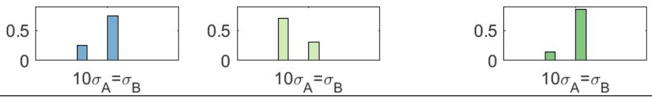		
-0.05	(0.006, 0.279)	η (0.011, 0.028) PD: 8.20, Size: 0.94, α : 0.816	η (0.004, 0.967) PD: 6.21, Size: 1.01, α : 0.184
	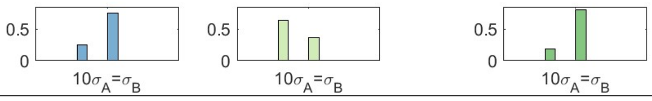		
-0.1	(0.006, 0.277)	η (0.011, 0.021) PD: 8.35, Size: 0.95, α : 0.818	η (0.003, 1.002) PD: 6.53, Size: 1.01, α : 0.182
	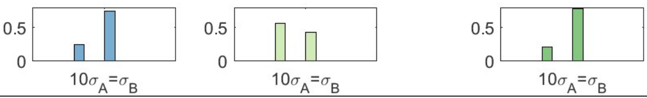		

Table S IV. Critical points for the different binary mixtures depending on the non-additivity of component B, and phase separated concentrations and volume fraction α of the different mixtures for specific parent concentration ($\eta_{A_{parent}} = 0.010, \eta_{B_{parent}} = 0.200$), depending on the non-additivity of component B, $PD = 6.93$

$\Delta_{B_aB_b}$	η_{crit}	**Top phase**	**Middle phase**	**Bottom phase**
0.100	-	η (0.011, 0.065)<br>PD: 3.05,<br>Size: 0.97,<br>α : 0.819	η (0.005, 0.643)<br>PD: 4.43,<br>Size: 0.97,<br>α : 0.097	η (0.002, 1.376)<br>PD: 2.88,<br>Size: 1.11,<br>α : 0.04
	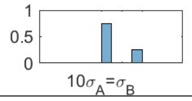	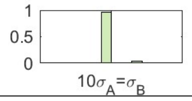	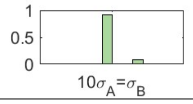	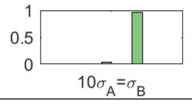
0.05	(0.006, 0.325)	η (0.011, 0.050)<br>PD: 2.96,<br>Size: 0.97,<br>α : 0.813		η (0.004, 0.854)<br>PD: 7.35,<br>Size: 1.01,<br>α : 0.187
	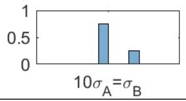	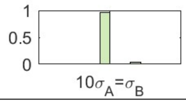		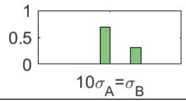
0	(0.006, 0.326)	η (0.011, 0.044)<br>PD: 2.36,<br>Size: 0.96,<br>α : 0.826		η (0.004, 0.941)<br>PD: 7.33,<br>Size: 1.01,<br>α : 0.174
	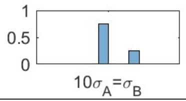	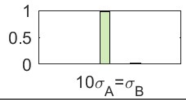		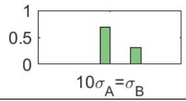
-0.05	(0.006, 0.331)	η (0.011, 0.040)<br>PD: 1.84,<br>Size: 0.96,<br>α : 0.837		η (0.003, 1.025)<br>PD: 7.31,<br>Size: 1.01,<br>α : 0.163
	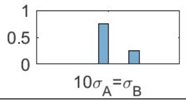	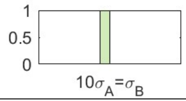		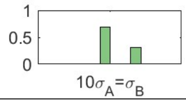
-0.1	(0.005, 0.337)	η (0.011, 0.037)<br>PD: 1.42,<br>Size: 0.96,<br>α : 0.848		η (0.003, 1.106)<br>PD: 7.29,<br>Size: 1.01,<br>α : 0.142
	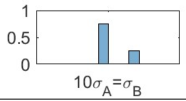	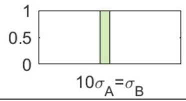		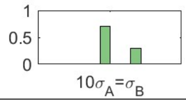

Table S V. Critical points for the different binary mixtures depending on the non-additivity of component B, and phase separated concentrations and volume fraction α of the different mixtures for specific parent concentration ($\eta_{A_{parent}} = 0.010, \eta_{B_{parent}} = 0.200$), depending on the non-additivity of component B, $PD = 4.80$

$\Delta_{B_aB_b}$	η_{crit}	**Top phase**	**Bottom phase**
0.100	(0.007, 0.295)	η (0.011, 0.039) PD: 8.51, Size: 0.94, α : 0.817	η (0.004, 0.918) PD: 1.77, Size: 1.01, α : 0.183
	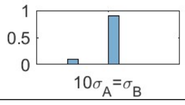	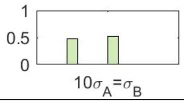	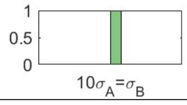
0.05	(0.007, 0.284)	η (0.011, 0.036) PD: 8.40, Size: 0.95, α : 0.811	η (0.004, 0.904) PD: 2.69, Size: 1.01, α : 0.189
	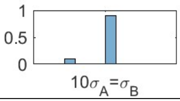	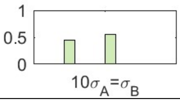	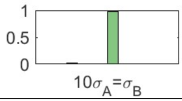
0	(0.007, 0.276)	η (0.011, 0.032) PD: 8.12, Size: 0.96, α : 0.805	η (0.004, 0.895) PD: 3.53, Size: 1.01, α : 0.195
	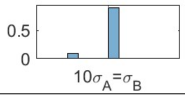	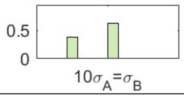	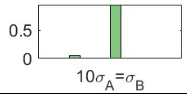
-0.05	(0.007, 0.271)	η (0.012, 0.028) PD: 7.58, Size: 0.97, α : 0.803	η (0.004, 0.899) PD: 4.11, Size: 1.00, α : 0.197
	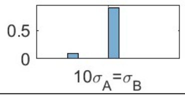	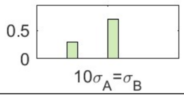	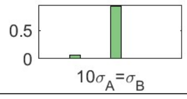
-0.1	(0.007, 0.270)	η (0.012, 0.025) PD: 6.79, Size: 0.98, α : 0.803	η (0.004, 0.914) PD: 4.46, Size: 1.00, α : 0.197
	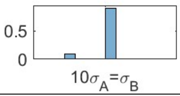	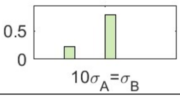	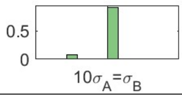

Table S VI. Critical points for the different binary mixtures depending on the non-additivity of component B, and phase separated concentrations and volume fraction α of the different mixtures for specific parent concentration ($\eta_{A_{parent}} = 0.010, \eta_{B_{parent}} = 0.200$), depending on the non-additivity of component B, $PD = 4.80$

$\Delta_{B_aB_b}$	η_{crit}	**Top phase**	**Middle phase**	**Bottom phase**
0.100	(0.007, 0.262)	η (0.011, 0.040)<br>PD: 2.47,<br>Size: 0.99,<br>α : 0.791	η (0.005, 0.767)<br>PD: 3.71,<br>Size: 0.99,<br>α : 0.198	η (0.002, 1.472)<br>PD: 2.57,<br>Size: 1.14,<br>α : 0.011
	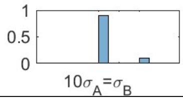	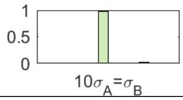	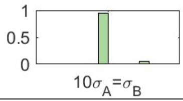	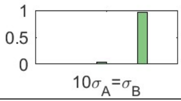
0.05	(0.007, 0.279)	η (0.011, 0.037)<br>PD: 1.90,<br>Size: 0.99,<br>α : 0.795		η (0.004, 0.832)<br>PD: 5.10,<br>Size: 1.00,<br>α : 0.205
	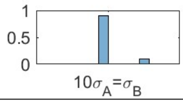	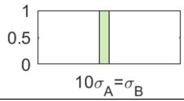		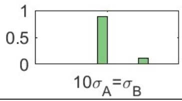
0	(0.006, 0.304)	η (0.011, 0.036)<br>PD: 1.20,<br>Size: 0.98,<br>α : 0.807		η (0.004, 0.885)<br>PD: 5.13,<br>Size: 1.00,<br>α : 0.193
	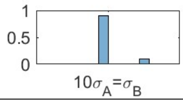	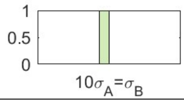		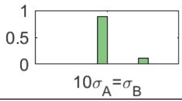
-0.05	(0.006, 0.335)	η (0.011, 0.036)<br>PD: 0.75,<br>Size: 0.98,<br>α : 0.818		η (0.004, 0.935)<br>PD: 5.15,<br>Size: 1.00,<br>α : 0.182
	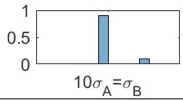	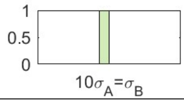		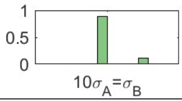
.-0.1	(0.006, 0.369)	η (0.011, 0.037)<br>PD: 0.47,<br>Size: 0.98,<br>α : 0.828		η (0.003, 0.982)<br>PD: 5.17,<br>Size: 1.00,<br>α : 0.172
	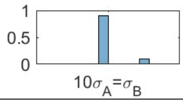	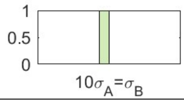		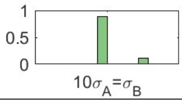
